# Tarantula welfare may be improved with greater environmental complexity: A preliminary behavioral study with Brazilian black tarantulas (*Grammastola pulchra*)

**DOI:** 10.1371/journal.pone.0314501

**Published:** 2024-12-05

**Authors:** Laura Stalter, Tayler Dorleus, Nicholas Milone, Jamie Sincage, Michelle Skurski, Austin Leeds

**Affiliations:** Animals, Science and Environment, Disney’s Animal Kingdom^®^, Lake Buena Vista, FL, United States of America; University of Illinois Chicago Department of Biological Sciences, UNITED STATES OF AMERICA

## Abstract

Increased environmental complexity has positive effects on the welfare status of vertebrates living in human care; however, this line of research has received little focus in invertebrates. Here we conducted a preliminary investigation of Brazilian black tarantula (*Grammastola pulchra*) behavior in relation to environmental complexity. Using a counterbalanced design, we housed tarantulas in both Standard and Complex environments. Complex housing was differentiated from Standard by being approximately six times larger and containing six times as many structural elements. We evaluated (1) how does tarantula behavior differ between these two housing conditions, (2) does housing affect tarantula behavior in a novel environment test, and (3) do tarantulas prefer one housing condition over the other? Activity budgets were approximately equivalent between the two housing conditions with inactivity accounting for nearly all observed behavior. Home ranges and core areas were 77% and 113% larger in the Complex housing condition. When in a novel environment, tarantulas had 150% greater odds of being active after living in the Complex condition. Interestingly, the tarantulas showed no clear preference for either environment in the preference test. Our preliminary results suggest tarantula welfare may be improved with greater environmental complexity as demonstrated through more neophilic response to novelty and greater home range and core area size when living in Complex housing. However, it is worth noting that broad patterns of behavior were similar, no clear preference in housing was identified, and our study sample size was limited. Further research is needed to better understand the behavior and welfare of tarantulas, but this study demonstrates how established methodologies used in the study of vertebrates can be readily applied to invertebrates.

## Introduction

Habitat complexity refers to the quantity and quality of biotic and abiotic components within ones living space [1]. For animals living in human care, this includes the amount of vertical and horizontal space, the density, diversity, and placement of structural elements (e.g., physical structures, plants, substrates), the presence of other animals within and adjacent to the habitat, and the sensory environment within the space (e.g., temperature, humidity, light cycle, sound landscape, exposure to visitors). In theory, complex habitats, often referred to as enriched environments, provide animals the opportunity to make more choices and face more challenges in their day-to-day lives than habitats that are less complex. These choices and challenges create opportunities for animals to maintain control, and ultimately agency, over their lives. This choice, challenge, control, agency progression is theorized to be a major contributor to an animal’s overall welfare status [2]. Thus, providing animals with appropriately complex habitats is considered a critical component of animal care. Findings to-date have shown that greater environmental complexity is associated with positive care and welfare outcomes including: increased species-typical behavior [3–15], reduced stereotypic behavior [9, 11, 15, 16], increased affiliative behavior [3, 10, 14, 17] decreased agonism [5, 18], improved reproductive success [4], expanded use of exhibit space [11, 12, 19], and improved physical appearance [3, 4].

Studies examining the effects of exhibit complexity on animals in human care have primarily focused on mammals [20, 21], but recent efforts have expanded this line of inquiry to non-mammalian taxa, including birds [22, 23], fish [11, 24] reptiles [4, 5, 8, 16, 25–29], amphibians [30], crustaceans [18], and nematodes [7]. These non-mammalian examples provide evidence that exhibit complexity is likely a valuable contributor to the care, and ultimately welfare, of all taxa. For example, providing Madagascar giant hognose snakes (*Leioheterodon madagascariensis*) with larger enclosures, open glass fronts, and naturalistic elements, including, deep sand/mulch substrate and corkbark furnishings, led to increased behavioral diversity and environmental exploration [31]. Similarly, Port Jackson sharks (*Heterodontus portusjacksoni*) had a reduced occurrence of stereotypic behavior, greater engagement in species-typical behavior, and greater use of space following an exhibit renovation that increased exhibit complexity via the addition of structural elements, hides, and a novel substrate [11].

Studies of the effect of environmental complexity on invertebrate welfare are rare despite being a commonly managed taxa in human care [18, 32]. One reason may be that their cryptic behavior is difficult to observe and interpret. Though an understudied topic, we argue that many of the “classic” approaches used in the study of vertebrates have value in the study of invertebrates as well. Evaluating the frequency of engagement in species-typical behavior is likely just as valuable for invertebrates as it is for vertebrates [4, 5, 8, 11]. Assessing space use patterns can be used to uncover changes in behavior, for example, by observing whether animals utilize new enclosure elements [19] or increase their distribution within an exhibit [12]. Preference testing can also be used to more explicitly identify individual preferences [33] and is becoming a more common approach in non-mammalian studies. For example, Hoehfurtner et al. (2021) found that corn snakes (*Pantherophis guttatus*) showed a preference for more complex environments over a less complex environment when given the choice [26]. A reduction in stereotypic behavior can also be informative in understanding how animals respond to complexity [34]. Interactions with a transparent boundary (ITB), where an animal repeatedly interacts with the wall of their housing space, is a common stereotypic behavior in reptiles associated with insufficient environmental complexity [35, 36]. This behavior has not yet been described in invertebrates; however, like reptiles, they are typically housed in bin/tank type enclosures with transparent boundaries and thus ITB or ITB-like behavior may be worthy of investigation in invertebrates.

Cognitive bias tests are another tool that have been used to assess how management practices affect animals by investigating markers of cognitive emotional states, also referred to as affects [37, 38]. The theory behind cognitive bias tests stems from human psychology and has been validated for use in multiple species [22, 37–46]. Generally, those with more pessimistic affects will judge an ambiguous stimulus with fear or anxiety, while those with more optimistic affects will judge the same ambiguous stimulus in a more relaxed, exploratory way [47, 48]. Novelty tests, where an animal’s response to an unfamiliar object or environment is observed [49–52], are a common form of cognitive bias testing. To-date, studies have found animals living in more complex environments behaved more neophilic than individuals living in less complex environments which preliminarily suggests complex environments may positively affect an animal’s affective state [27, 53–55]. This experimental design is beginning to be used with invertebrates, for example, Liedtke et al. [56] observed that jumping spiders (*Marpissa muscosa*) raised in a complex environment explored more during a novel environment test than those raised in a less complex environment. Similarly, Bengston et al. [57] found that providing tarantulas (*Brachypelma* spp.) a naturalistic substrate and artificial plant reduced retreat behavior to experimental prodding, decreased latency to attack prey, and increased locomotion in a novel environment compared to tarantulas living without these environmental features.

In human care, arachnids are typically housed in relatively simple environments, and concerns have been raised suggesting their care and management is in need of greater attention [58]. At Disney’s Animal Kingdom®, Brazilian black tarantulas (*Grammastola pulchra*) are managed in an off-exhibit building and housed in containers 27x27x21 cm in size with substrate 2.5 cm deep, furnished with a single piece of cork bark and a water dish. We were interested in evaluating how these tarantulas would respond to a more complex housing environment.

Brazilian black tarantulas are a member of the Theraphosidae family [59], are popular in both zoological populations and the pet trade, and are not well studied in- or ex-situ. They are a terrestrial species that typically live in burrows that provide protection from predators and parasites, especially during molting and reproduction [59, 60]. Most Theraphosid tarantulas are nocturnal sit-and-wait predators [60]. Adult females will remain in or near their burrow, emerging to feed at night while males show more exploratory behavior [60]. This increased exploratory behavior, expressed primarily via locomotor activity, in males has been described as a seasonal behavioral pattern associated with breeding [e.g. 61]. How male and female behavior differs outside of this seasonal period is less known, as is how these behavioral differences manifest in human care.

The purpose of this study was to evaluate the behavioral response of Brazilian black tarantulas to increased environmental complexity. We studied the behavior of tarantulas in two conditions–a Standard housing condition (i.e., a replication of their existing housing space) and a Complex housing condition (i.e., a larger and more physically complex housing space). In addition to behavior monitoring in both housing conditions, we utilized novelty and preference tests to further evaluate their response to these environments. Specifically, we sought to answer:

1. Do species-typical behavior, space use patterns, and/or physical attributes differ between housing conditions?
2. Do responses to novelty differ between housing conditions?
3. Do tarantulas exhibit a preference for either housing condition?

By utilizing both behavioral observations and experimental tests on behavior, we hoped to gain a better understanding of the impact environmental complexity has on tarantula behavior and ultimately welfare. We hypothesized that providing a more complex space will increase species-typical behavior of Brazilian black tarantulas and will result in reduced neophobic behavior, an increase in space use, and a clear preference shown for the more complex environment. To our knowledge, this has not been evaluated in Brazilian black tarantulas to date, so findings from this study should be informative for their care and management. More generally, we also hope this will highlight the value and need for studying invertebrate behavior, care, and welfare more broadly.

## Methods

### Ethical note

This study was conducted with approval of the Scientific Review Committee and Animal Care and Welfare Committee of Disney’s Animals, Science and Environment^®^.

### Study subjects and housing

Fifteen (n_male_ = 4; n_female_ = 11) Brazilian black tarantulas participated in this study. Individuals lived in an off-display invertebrate management building at Disney’s Animal Kingdom^®^ (Lake Buena Vista, Florida, USA). All tarantulas were acquired from a government confiscation, thus full demographic history of individuals is unknown. Prior to this study, tarantulas were singly housed in a clear plastic container 27x27x21 cm in size that contained 2.5 cm of dirt substrate, a piece of cork bark, and a water dish. Building temperature was maintained between 25.6 and 26.7°C. Light was provided by overhead LED light fixtures on a 10.5/13.5hr light/dark cycle.

**Fig 1:**
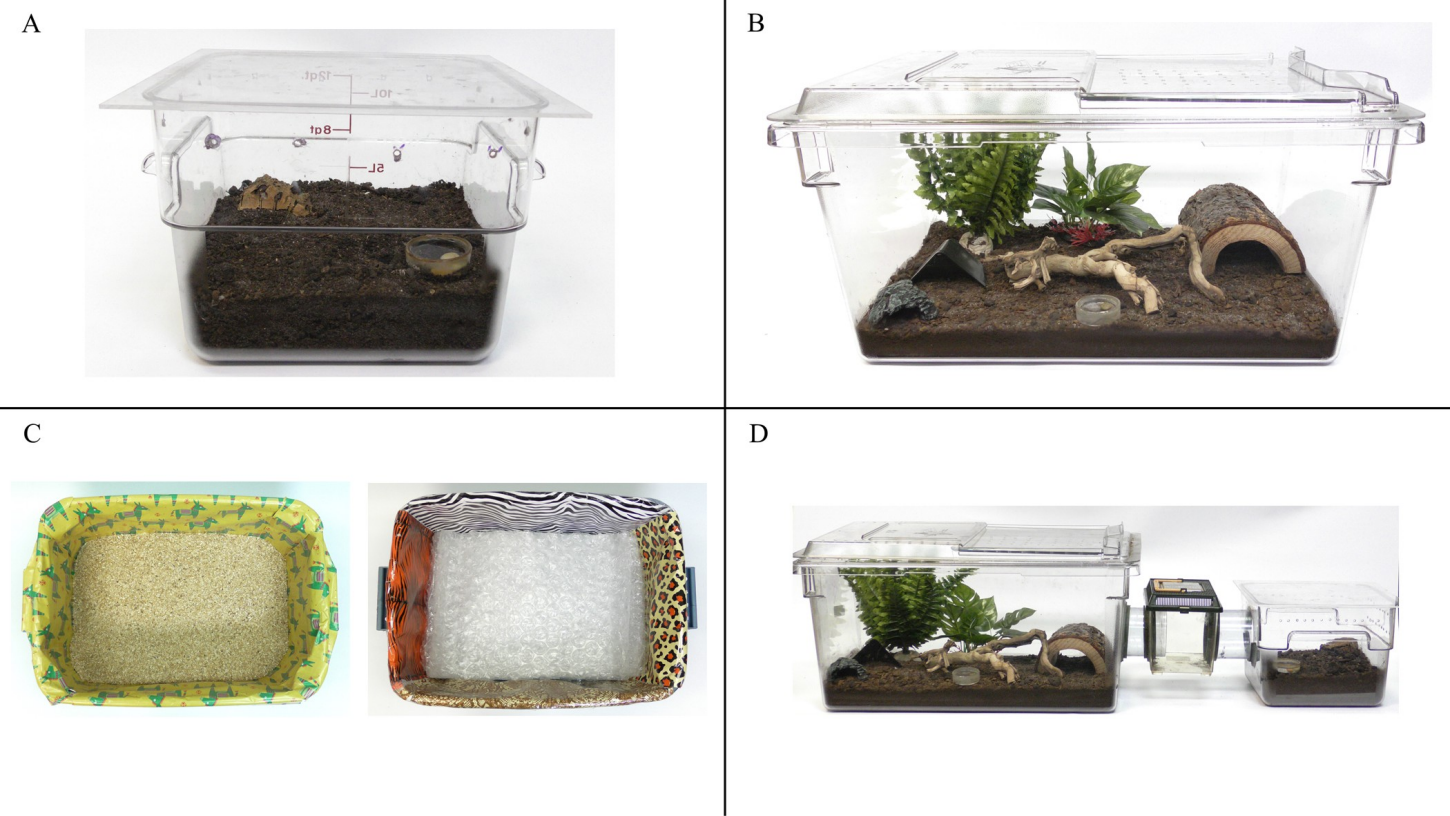
Photographs of the study housing: (A) Standard housing, (B) Complex housing, (C) the two novel environment test apparatuses, (D) preference test apparatus.

For the study, tarantulas were housed in two enclosure types. The Standard housing condition was a replication of the tarantulas’ typical housing described previously but was not the same enclosure they had been previously living in. For the Complex housing condition, tarantulas were housed in a clear plastic Rubbermaid^®^ 22 gallon Cambro container 71x41x31 cm in size. The enclosure contained 2.5 cm of dirt substrate, a water dish, and six enclosures elements: two plastic plants, a dead fall branch, a small plaster rock cave, a plastic garden pot placed upside down with an opening cut out on one side, and a large U-shaped wooden hide (see Fig 1 for images of both housing conditions). Tarantulas were housed in each enclosure for four weeks. Order of enclosure presentation was counterbalanced across subjects (see S1 Fig in S3 File for visual diagram of experimental set up). Study enclosures were managed in the same building as they normally lived and thus light cycle and ambient temperatures remained consistent.

### Comparing housing conditions

#### Activity budget

Once placed in a study enclosure, tarantulas were provided four days to acclimate prior to the initiation of the four-week observation period. Tarantula behavior was recorded via Yeskamo wireless home security cameras mounted in front of each enclosure. Ten-minute behavior observations were conducted on each tarantula twice a day, six days per week. Observations were balanced across each hour of the day, totaling eight hours of observation per individual per enclosure type (256 total observation hours across all subjects/conditions). Activity was collected via scan sampling at 30 sec intervals. In addition, the incidence of a tarantula falling from a vertical location was recorded via all occurrence sampling. Falling was incorporated into this study due to concerns of complex environments increasing the risk of injury to residents. The activity ethogram can be found in Table 1, which was developed via a pilot observation period of the tarantulas prior to start of the study. Authors LS and AL coded video for this study. Interobserver reliability was established by review and discussion of initial video examples of behavior, followed by the completion of eight observations for which >85% agreement for each behavior category was established.

**Table 1:** Ethogram and activity budget by housing condition and time of day. Values rounded to three decimal places, anything below three decimal places are rounded to 0.000. Unobserved behaviors are noted as such. Mean difference was calculated by subtracting Complex housing value from Standard housing value.

Behavior and Definition	Mean Proportion of Time or Mean Rate per Observation					
	Dark Hours			Light Hours		
	Standard Housing (SD)	Complex Housing (SD)	Mean Difference (95% CI)	Standard Housing (SD)	Complex Housing (SD)	Mean Difference (95% CI)
***Inactive***	0.952 (0.048)	0.970 (0.029)	-0.018 (0.035)	0.972 (0.017)	0.969 (0.037)	0.004 (0.040)
Tarantula is not engaged in any active behavior. Output is mean proportion of time.						
***Locomotion***	0.009 (0.010)	0.009 (0.008)	0.000 (0.007)	0.004 (0.004)	0.003 (0.005)	0.000 (0.007)
Tarantula moves >50% of body in a direction away from their original location with body horizontal to the ground. Output is mean proportion of time.						
***Climb***	0.018 (0.020)	0.009 (0.011)	0.009 (0.014)	0.010 (0.009)	0.005 (0.009)	0.005 (0.010)
Tarantula locomotes with head going in a vertical direction (up or down) or more than 50% of the tarantula is on the wall/furniture item locomoting. Body may be perpendicular to the ground. Output is mean proportion of time.						
***Active in Place***	0.015 (0.019)	0.009 (0.013)	0.006 (0.018)	0.010 (0.010)	0.020 (0.032)	-0.009 (0.035)
One or more legs are in motion but tarantula remains in place. May include tapping. Output is mean proportion of time.						
***Preen***	0.003 (0.006)	0.003 (0.009)	0.000 (0.011)	0.001 (0.002)	0.002 (0.004)	-0.001 (0.004)
Tarantula passes legs or pedipalps through the chelicerae. Output is mean proportion of time.						
***Web Construction***	0.003 (0.006)	Unobserved	0.003 (0.006)	0.002 (0.006)	0.001 (0.004)	0.001 (0.007)
Both spinnerets remain parallel while moving together in a rhythmic pattern. Abdomen of tarantula moves up and down with spinneret movement. Often accompanied by leg movement and shifts in the tarantula’s position. Output is mean proportion of time.						
***Dig/Burrow***	Unobserved	Unobserved	Not Applicable	0.001 (0.002)	Unobserved	0.001 (0.002)
Tarantula moves and/or manipulates substrate or webbing with appendages. May include web production. Output is mean proportion of time.						
***Leg Raise***	Unobserved	Unobserved	Not Applicable	Unobserved	Unobserved	Not Applicable
Tarantula raises pedipalps and front legs 1 and 2 off ground. Output is mean proportion of time.						
***Fall***	0.023 (0.218)	0.003 (0.054)	-0.022 (0.068)	0.015 (0.173)	0.014 (0.116)	0.004 (0.056)
Tarantula exhibits a rapid downward movement of at least half a body length without control. Output is mean rate per observation.						
***Other***	0.000 (0.000)	Unobserved	0.000 (0.000)	Unobserved	Unobserved	Not Applicable
Tarantula is engaged in a behavior not listed on this ethogram. Output is mean proportion of time.						

#### Space use

During each ten-minute behavior observation, the tarantula’s location was recorded at two-minute intervals on a map depicting both the horizontal and vertical planes of the enclosure in the ZooMonitor application [62]. ZooMonitor uses a 600x600 grid, and these grid locations were imported into ArcGIS Pro. To account for the entire space, the locations along the wall were edited while maintaining the same distance from the ground to create a single 2-dimensional diagram of the enclosure. Wall locations were then georeferenced to the new 2-dimensional diagram using a rubbersheet (natural neighbor) transformation to ensure the most accurate locations.

Home ranges (HR) and core areas (CA) are commonly used in wildlife ecology to describe animal space use. These methods have recently started to be adapted for use in zoo environments [63, 64] and here we utilize home ranges and core areas to quantify how much space the tarantulas use within their enclosures. In ArcGIS Pro, kernel density estimates were created for each tarantula in each housing condition using the enclosure borders as a barrier and bandwidth values determined by the optimal bandwidth function,

$$h_{opt}={\left[\frac{2}{3n}\right]}^{\left(\frac{1}{4}\right)}\sigma ,$$

where *n* is the number of locations and σ is the standard distance of locations [65]. We used the 95% fixed-kernel method to define the home range, which encompasses most of the locations recorded and therefore the general area that the tarantula has been observed, as well as the 50% fixed-kernel method, which quantifies highly concentrated areas of use and can be interpreted as where the tarantula spends the most time. We calculated the proportion of the enclosure each home range and core area encompassed in ArcGIS Pro. We then calculated the true size of each home range and core area using these proportions and the true surface area of the enclosures. During preliminary analyses, we found the tarantulas spent most of their time on the ground and walls so we felt home ranges and core areas along the surface area of each enclosure would provide an accurate description of space used.

During each ten-minute observation, the tarantula’s use of enclosure elements (ground, wall, ceiling, and furniture) were also collected at 30 sec intervals. When under a hide or on furniture, the element in use was specified. To describe any potential habitat preferences in the Complex housing conditions, we divided the space into 7 zones, categorized by the enclosure elements contained within the zone or as the wall (S2 Fig in S3 File). We used an electivity index (E*) to compare the actual use of a zone to its expected use based on the zone’s size. Zones used in greater proportion than expected are considered preferred. The proportion of each zone was calculated in ArcGIS Pro and each tarantula location was assigned a zone using a spatial join. We used Vanderploeg and Scavia’s electivity index [66]:

$$E*=\frac{W_{i}-\left(\frac{1}{n}\right)}{W_{i}+\left(\frac{1}{n}\right)}$$

where *n* is the number of zones, and

$$W_{i}=\frac{\frac{r_{i}}{p_{i}}}{\sum \frac{r_{i}}{p_{i}}}$$

where r_i_ is the observed use (proportion of locations) of zone i and p_i_ is the expected use (proportion of locations) of zone i [63, 67, 68]. E* was determined for each tarantula to get an average E* value for each zone. We did not investigate preferences in the Standard housing condition because there were so few possible zones that we did not think it would provide any additional insight.

To further describe the tarantulas’ use of space related to hide and furniture use, we categorized each location within a zone as either In Hide, On Furniture, or on Open Space. We then calculated the mean proportion of locations per individual to get an average for each zone by usage category. The ethogram for defining how a tarantula utilized each enclosure feature and vertical level can be found in S2 Table in S2 File.

#### Feeding and body weight

Tarantulas were fed one commercially raised house cricket (*Acheta domesticus*) once a week. Feeding outcomes were recorded as either the tarantula did eat or did not eat (i.e. cricket was uneaten after 24 hours and removed). Body weight (g) was recorded at the start and end of each housing condition.

### Novel environment test

Following each housing condition described above, each tarantula participated in a novel environment test. The design was modeled after those described in Moszuti et al. [39]. Both were rectangular in shape (56x38x23 cm) and had a clear acrylic sheet as a top to contain tarantulas inside while allowing for observation. Environment A had a ground substrate composed of folded sheets of bubble wrap and each wall was covered in a different style of animal print themed wrapping paper. Environment B had a ground substrate of sawdust and the walls were covered in a brightly colored wrapping paper with cartoon animals (see Fig 1 for images of both test environments). For the wrapping paper, color vision in tarantulas has recently been described [69], though the overall quality of their vision is still in question. Thus, the color component of the wrapping paper may have fallen short of novel. However, preliminary observations of these tarantulas revealed they frequently spent time in contact with the walls of their enclosure. We felt the give/bend and vibration the wrapping paper made when contacted, in addition to being a smooth waxy surface, created a novel sensory experience for the tarantula and thus warranted inclusion in the novelty test. Following each four-week housing condition, a tarantula was placed in the center of one novel environment (counterbalanced across participants) for 10 minutes. Tarantulas were filmed via camcorder (Panasonic HC-VX981) during these tests. Behavior (using activity budget ethogram) and location (open space vs. contact with wall) were recorded via scan sampling at 15 sec intervals.

### Enclosure preference test

Once each tarantula had been housed in both enclosure types and participated in both novel environment tests, they participated in an enclosure preference test. The apparatus for this test was a replication of both the Standard and Complex housing conditions connected to each other via the short side of each enclosure. The two enclosures were connected via a clear acrylic tube (10 cm diameter, 20 cm in length) leading out of each enclosure to a small clear plastic terrarium (15x23x17 cm) centered between each enclosure. The terrarium contained no substrate, furniture, or water source. Tarantulas were placed in the center terrarium and then had free access between both the Standard and Complex housing conditions (see Fig 1 for image). Once placed in the apparatus, tarantulas were filmed for 24 hours via security cameras. First enclosure choice (Standard or Complex), frequency of entrance to each housing condition, and time (sec) in each enclosure were recorded.

### Data analysis

Generalized linear mixed models were used to model differences in the tarantulas’ behavior, space use, feeding outcome, and weight between conditions via the glmmTMB package [70, 71] in RStudio [72, 73]. Each dependent variable was modeled in relation to two fixed factors: housing, to evaluate experimental differences, and sex, to account for sex differences in behavior, as there is some data to suggest this may occur in tarantulas [60, 61]. Tarantula ID and observation date were included as random factors to account for individual variation and variation within observations conducted the same day, with the exception of HR and CA models. HR and CA models only included tarantula ID as a random factor, as HR and CA data are summarized for the entire condition irrespective of day. For behavioral dependent variables, as well as furniture use and wall use, we additionally ran separate models for observations conducted during dark (1700–600) and light (700–1500) time cycles. Tarantulas are nocturnal and we thus anticipated behavior to be expressed differently in these periods. Lighting was not consistent during the 1600 hour due to variation in husbandry team activity, so all observations from this hour were removed. Model fit was assessed by visual inspection of the residuals via QQ plots. All models expressed normal distribution of residuals unless noted in the results section. Multicollinearity within models was assessed using a variance inflation factor (VIF) testing via the vif function. All fixed factors had a VIF factor of ≤ 2.1, which was our cutoff to suggest multicollinearity was not present [74]. All behavior-based models, as well as feeding outcome models, furniture use models, and wall use models, were fit with a binomial distribution as data were processed as a proportion of visible scans per observation. For weight, HR size, and CA size, models were fit with a Gaussian structure. Hide use was not modeled as hides were absent in the Standard condition. Hide use data from the Complex condition are presented descriptively.

Model outputs were explored by evaluating estimated marginal means (EMM) of the dependent variables. We additionally report 95% confidence intervals (CI) for all estimates for increased contextualization and to describe uncertainty. We avoid reporting dichotomous significance values and instead report effect sizes between fixed factors to better contextualize and describe our data [75, 76]. Specifically, we present EMM ratios and odds ratios (OR), alongside their respective 95% CI, to describe the magnitude of difference between our fixed factors. Graphical representations of the raw data are presented alongside our modeled data for full transparency in data presentation [75–78].

Several models had skewed residuals (described in results). Attempts to normalize were unsuccessful and thus data are presented descriptively. Mean values (± standard deviation) are presented in these cases as well as in describing hide use and behavior during the preference test in which models were not conducted. To describe the magnitude of difference between conditions (where appropriate), paired mean differences (with 95% CI) were calculated using the meanDiff function of the package “rosetta” [79].

### Subject removal

Several tarantulas were excluded from some analyses or the study altogether. Explanation for each removal can be found in S1 Table in S2 File. Final sample sizes for comparing housing condition, novel environment test, and preference test analyses were 13 (n_females_ = 10), 9 (n_females_ = 8), and 13 (n_females_ = 10), respectively.

## Results

### Comparing housing conditions

#### Activity budget

Data are presented descriptively due to poor residual fit. The tarantulas spent most of their time inactive, regardless of time of day or housing condition (Table 1 and S3 Fig in S3 File). During dark hours, the mean proportion of time spent inactive in the Complex housing was 0.018 (SD = 0.035) greater than the Standard housing condition. Differences in mean proportion of time inactive during light hours approached zero (μ_difference_ = 0.004, SD = 0.040). Proportion of time devoted to other behaviors described in the ethogram can be broadly described as negligible (S4-S10 Figs in S3 File). Similar to housing condition, differences in behavior by sex by condition were limited (S3 and S4 Tables in S2 File). Falls were infrequent but occurred more often in the Standard housing condition. During dark hours the mean fall rate per observation was approximately 7.5x greater in the Standard housing condition than the Complex housing (μ_difference_ = 0.022, SD = 0.068). Fall rates during light hours were approximately equal between conditions (μ_difference_ = 0.004, SD = 0.056). Overall, falls were infrequent with only four individuals observed to fall regardless of housing condition.

#### Space use

**Fig 2:**
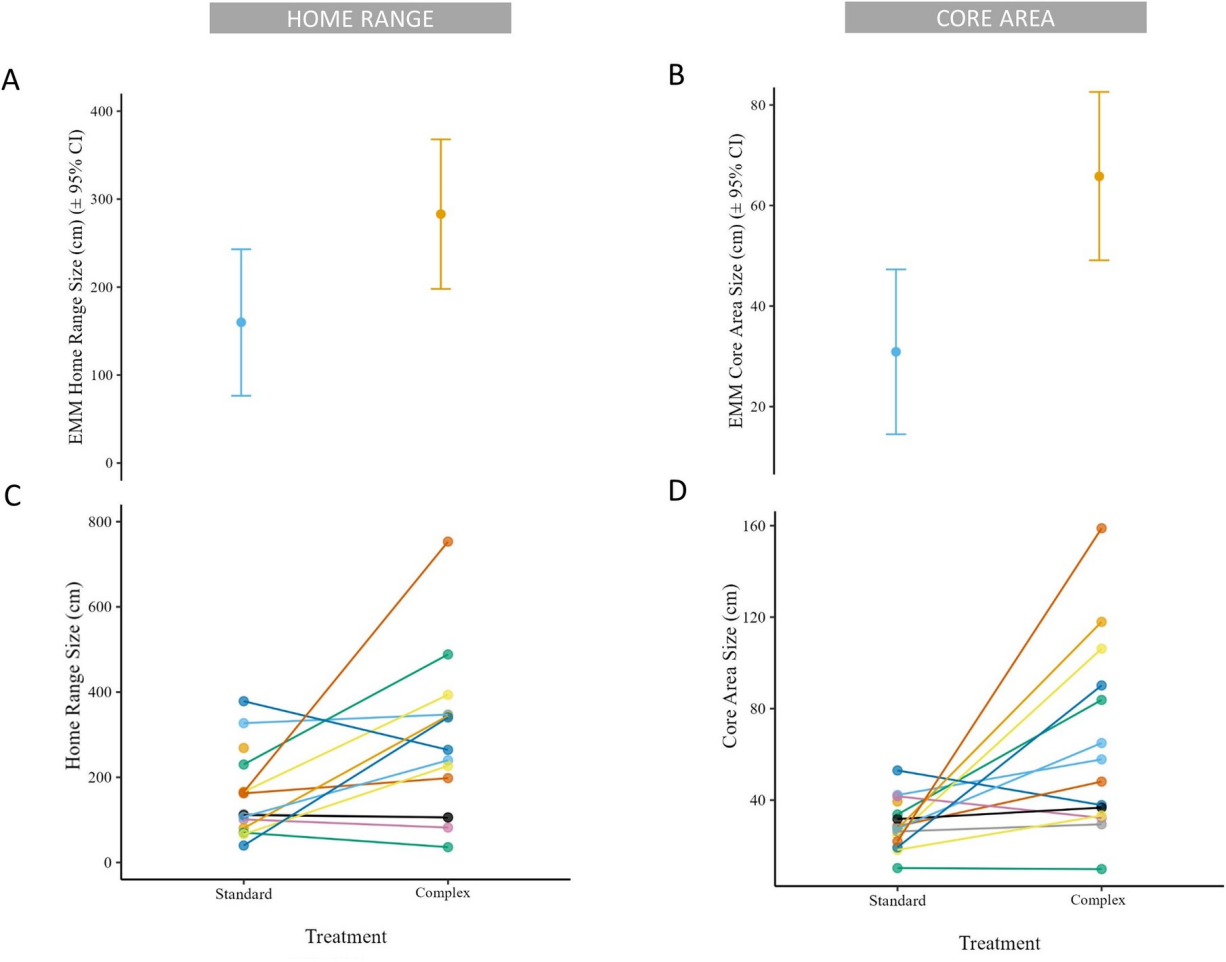
A&B: estimated marginal mean (EMM) of home range size (A) and core area size (B) by housing condition with 95% confidence intervals. C&D: individual home range size (A) and core area size (B) by housing condition. Paired samples for each tarantula are connected by line and labeled by unique color.

**Fig 3:**
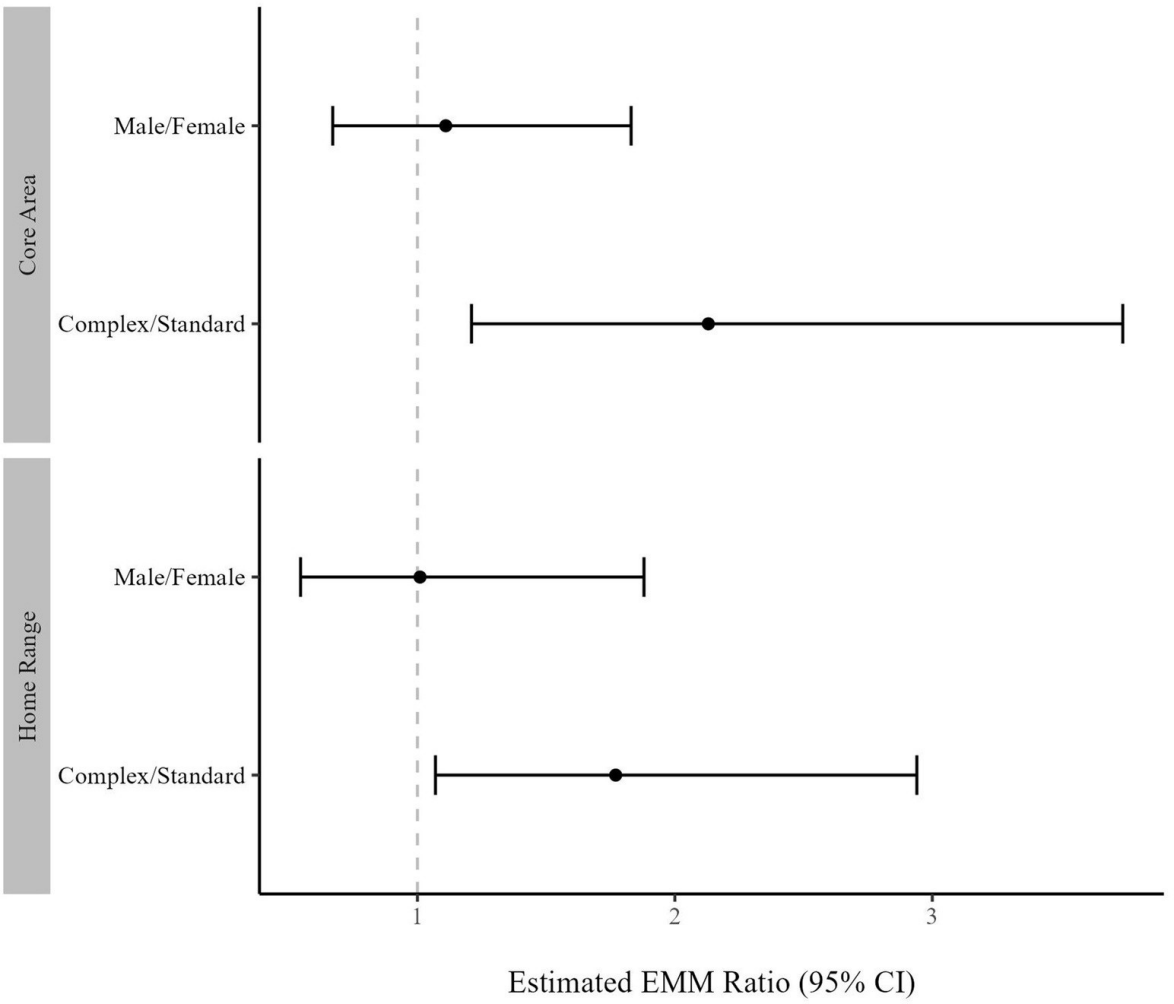
EMM ratios of individual home range and core area sizes by treatment and sex, bounded by 95% confidence intervals (CI). X axis value reflects relationship of numerator to denominator on Y axis label. Dashed grey line represents ratio of 1, or no difference.

Both HR and CA sizes were greater in the Complex housing condition than the Standard housing condition (Fig 2). On average, a tarantula’s HR was 77% larger (EMM_ratio_ = 1.77, CI: 1.07, 2.94) in the Complex housing condition (EMM = 283.0, CI: 197.9, 368.0) than the Standard housing condition (EMM = 160.0, CI: 76.5, 243.0; Fig 3; S5 Table in S2 File). A tarantula’s CA was 113% larger (EMM_ratio_ = 2.13, CI: 1.21, 3.74) in the Complex housing condition (μ_EMM_ = 65.8, CI: 49.1, 82.6) than the Standard housing condition (EMM = 30.9, CI: 14.5, 47.3; Fig 3 and S6 Table in S2 File). There was little evidence for a relationship between HR or CA size by sex (S11 Fig in S3 File; S5 and S6 Tables in S2 File).

Tarantulas utilized hides when they were available in the Complex housing condition. On average, the proportion of time tarantulas utilized hides in the Complex housing condition was 0.26 (SD = 0.30) at night and 0.29 (SD = 0.45) during the day (S12 Fig in S3 File). Limited evidence for a relationship between hide use and sex was observed at night (M_female_ = 0.14, SD = 0.34; M_male_ = 0.11; SD = 0.31) or during the day (M_female_ = 0.14, SD = 0.34; M_male_ = 0.16, SD = 0.37) (S12 Fig in S3 File). Similarly, the tarantulas’ use of furniture was approximately equal by housing condition (S13 Fig in S3 File) and sex (S14 Fig in S3 File).

**Fig 4:**
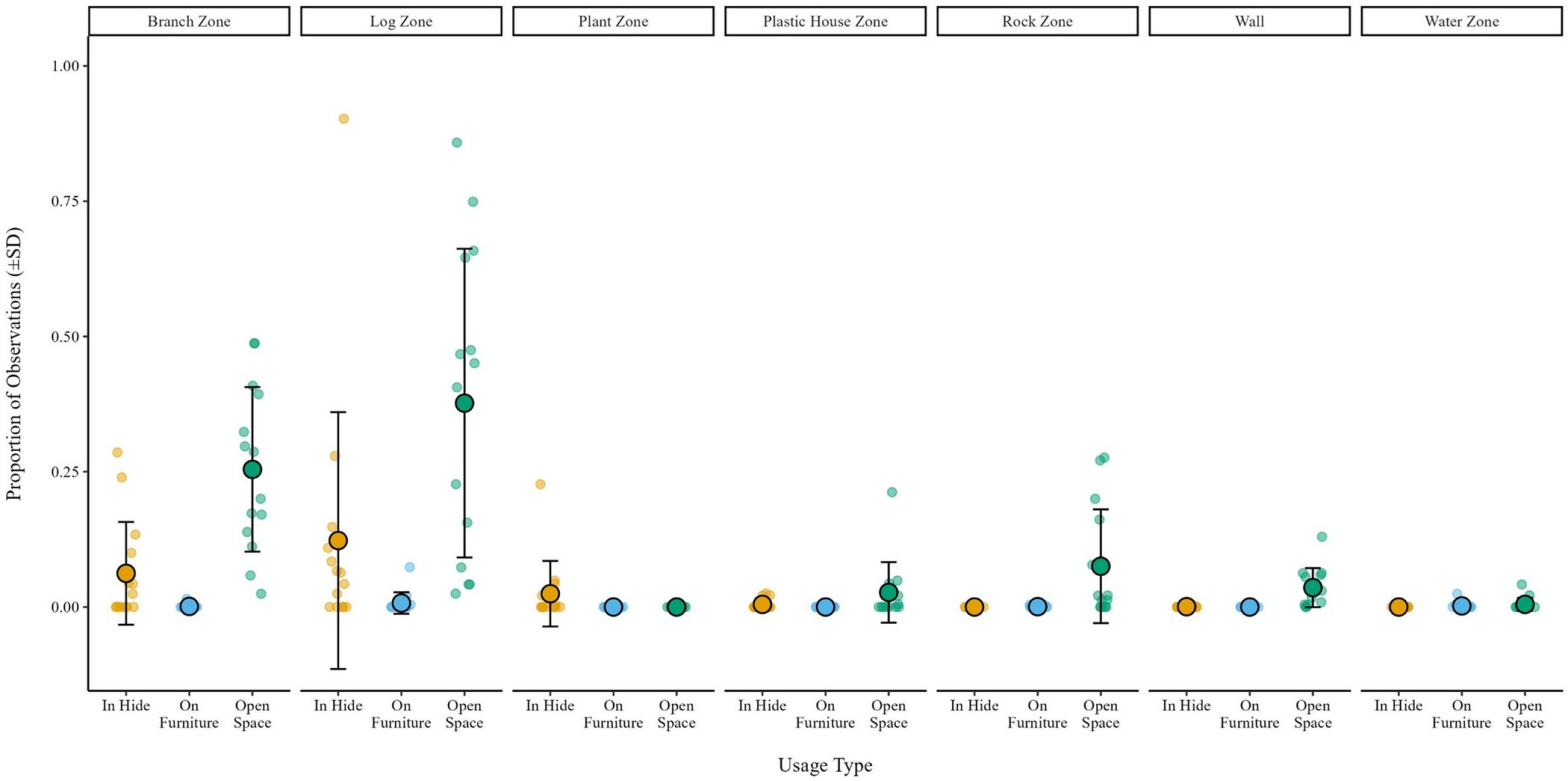
Proportion of observations by usage type (i.e. In Hide, On Furniture, Open Space) per individual by zone displayed via dot plot for all observations (n = 294). Larger circles outlined in black represent the mean proportion of observations, bounded by lines representing standard deviation.

In the Complex housing condition, the tarantulas utilized the habitat zones and furniture at different rates. On average, the tarantulas exhibited a preference for the Branch (E* = 0.14, SD = 0.34) and the Log Zone (E* = 0.27, SD = 0.49) in the Complex housing condition (S15 and S16 Figs in S3 File). Within both zones the tarantulas spent a higher proportion of observations in open space near the branch or log, respectively, rather than using them directly as hides (M_branch_ = 0.25, SD = 0.15; M_log_ = 0.38, SD = 0.29). When in a hide, the tarantulas spent the highest proportion of time under the log (M = 0.12, SD = 0.23), followed by the branch (M = 0.06, SD = 0.10). Time spent on top of furniture items approached zero (Fig 4).

**Fig 5:**
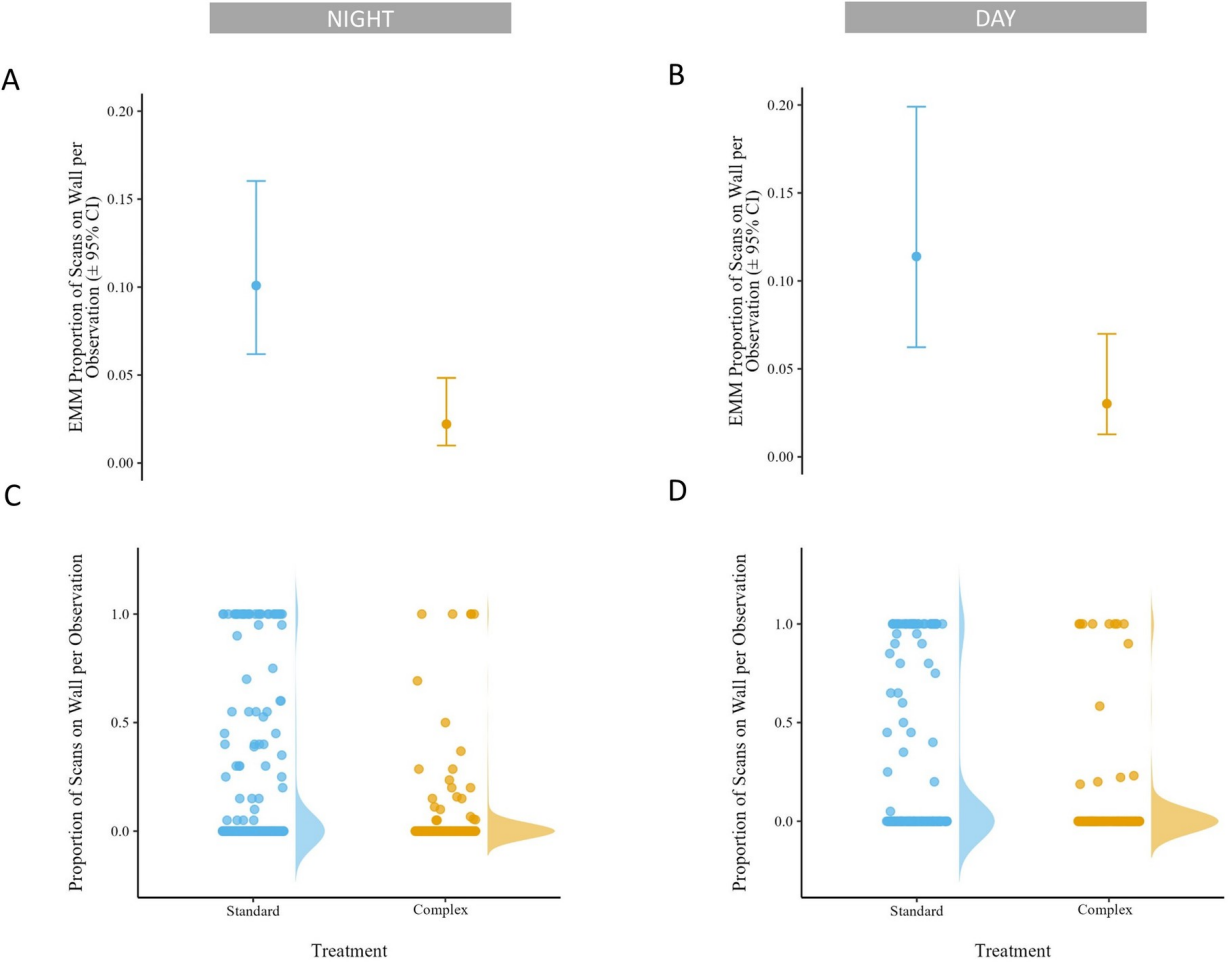
A&C: estimated marginal mean (EMM) of proportion of scans on wall per observation by housing condition bounded by 95% confidence intervals (CI) at night (A) and during the day (C). B&D: proportion of scans on wall per observation by housing condition at night (B) and during the day (D) displayed via dot plot and distribution curve for all observations (n_night_ = 739, n_day_ = 486).

**Fig 6:**
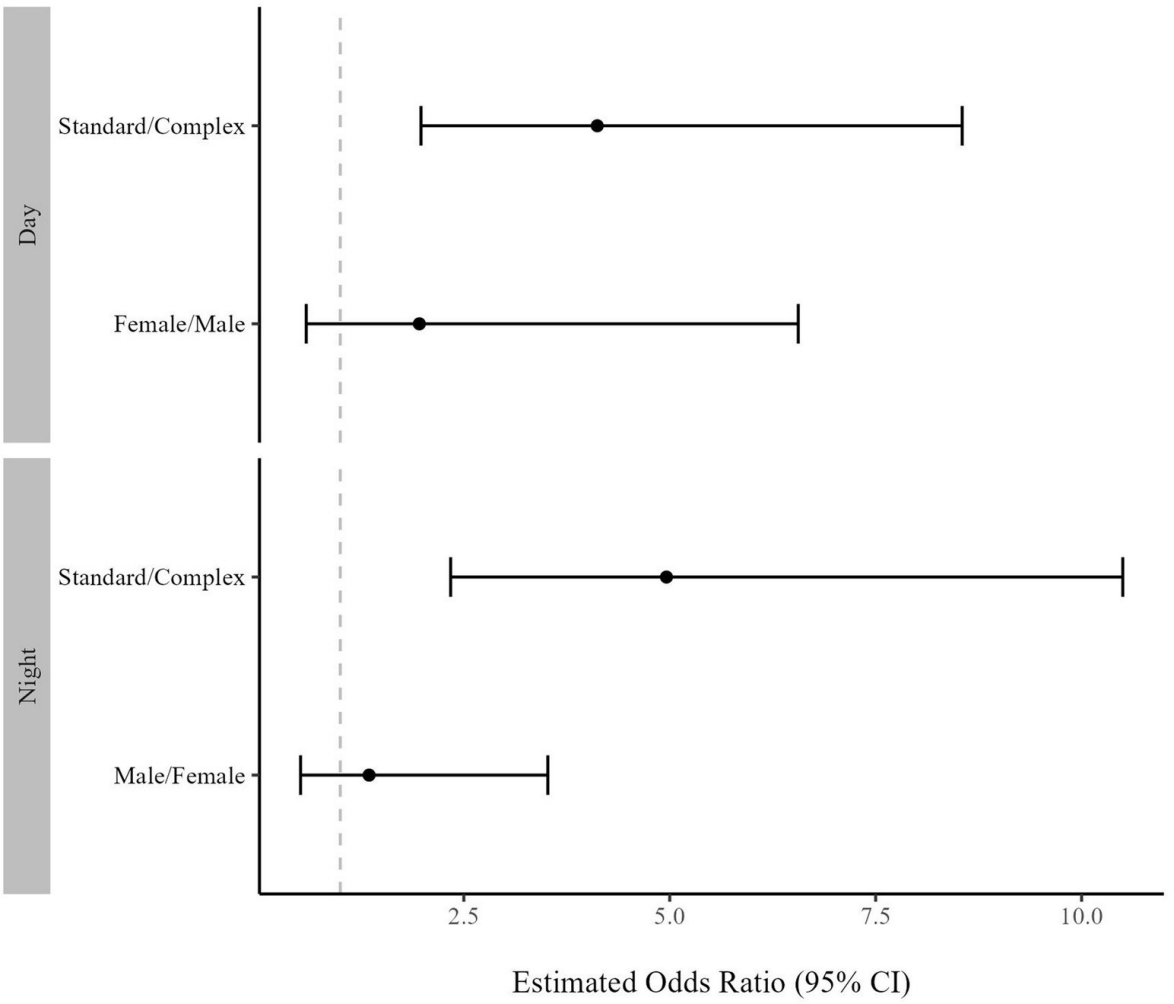
Odds ratios of scans on wall per observation for treatment and sex at night and during the day, bounded by 95% confidence intervals (CI). X axis value reflects relationship of numerator to denominator on Y axis label. Dashed grey line represents ratio of 1, or no difference.

Tarantulas were on the wall of their habitat more in the Standard housing condition than the Complex housing condition (Fig 5; S7 and S8 Tables in S2 File). A tarantula had 396% greater odds of being on the wall (OR = 4.96, CI: 2.34, 10.5) at night and 312% greater odds (OR = 4.12, CI: 1.98, 8.55) during the day in the Standard housing condition (EMM_night_ = 0.10, CI: 0.06, 0.16; EMM_day_ = 0.11, CI: 0.06, 0.12) than the Complex housing condition (EMM_night_ = 0.02, CI: 0.01, 0.05; EMM_day_ = 0.03, 0.01, 0.07; Fig 6). Broad confidence intervals suggested limited evidence of a relationship between wall use and sex during the day (EMM_female_ = 0.08, CI: 0.05, 0.14; EMM_male_ = 0.04, CI: 0.01, 0.12; OR = 1.96, CI: 0.59, 6.56) or at night (EMM_female_ = 0.04, CI: 0.02, 0.07; EMM_male_ = 0.06, CI: 0.02, 0.12; OR = 1.35, CI: 0.52, 3.25; S17 Fig in S3 File; S7 and S8 Tables in S2 File).

#### Feeding and body weight

The proportion of feeds in which a tarantula did not eat was low and did not differ between housing conditions (EMM_standard_ = 0.06, CI: 0.006, 0.39; EMM_complex_ = 0.06, CI: 0.006, 0.39; S18 Fig in S3 File; S9 Table in S2 File). Sex differences in the proportion of non-feeding days was also limited (EMM_female_ = 0.03, CI: 0.004, 0.26; EMM_male_ = 0.09, 0.01, 0.53; S18 Fig in S3 File; S9 Table in S2 File). Weight did not differ between housing conditions (EMM_standard_ = 18.50, CI: 16.10, 20.80; EMM_complex_ = 18.50, CI: 16.20, 20.80; S19 Fig in S3 File; S10 Table in S2 File). While females had a wider range of weights compared to males, the EMM difference was small with a broad CI suggesting limited effect of sex on weight (EMM_difference_ = 2.27, CI: -2.34, 6.87; EMM_female_ = 19.60, CI: 17.20, 22.0; EMM_males_ = 17.30, CI: 13.40, 21.30; S19 Fig in S3 File, S10 Table in S2 File).

### Novel environment test

**Fig 7:**
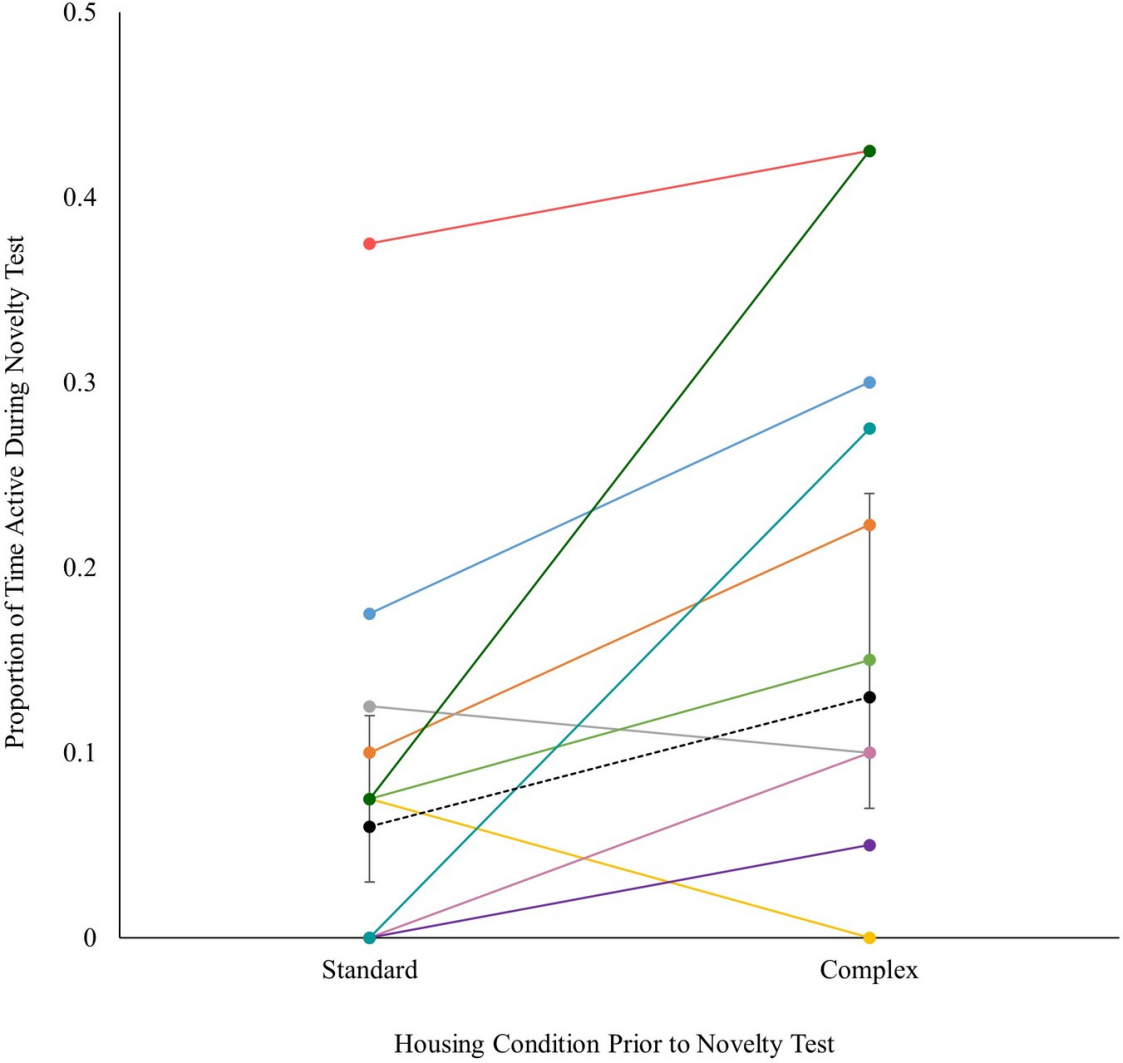
Proportion of scans active during novelty tests by housing condition. Paired samples for each tarantula are connected by line and labeled by unique color. Black circles connected by dashed line represent modelled EMM values, bound by 95% confidence intervals.

Tarantulas were more neophilic following their time in the Complex housing condition. During the test, a tarantula had 150% greater odds (OR = 2.50, CI: 1.63, 3.84) of being active following their time in the Complex housing condition (EMM = 0.13, CI: 0.07, 0.24) than the Standard housing condition (EMM = 0.06, CI: 0.03, 0.12; Fig 7 and S11 Table in S2 File). The residuals for the time in contact with the wall model were heavily skewed, thus a descriptive analysis of these data are presented. Mean time spent in contact with the wall was approximately equal between conditions (M_standard_ = 0.62, SD = 0.45; M_complex_ = 0.56, SD = 0.37; M_difference_ = 0.05, CI: -0.33, 0.44; S20 Fig in S3 File).

### Enclosure preference test

Tarantulas did not show a clear preference for one environment over the other. One tarantula (female) did not leave the selection chamber during the testing period. Of those that did leave the selection chamber, 50% (n_female_ = 5; n_male_ = 1) entered the Standard housing first and the other 50% (n_female_ = 4; n_male_ = 2) entered the Complex housing first. Overall, the proportion of time tarantulas spent between the two housing conditions was approximately equal (M_standard_ = 0.53, SD = 0.47; M_complex_ = 0.47, SD = 0.47; M_difference_ = 0.05, CI: -0.58, 0.68). Half of the tarantulas (n_female_ = 5; n_male_ = 1) entered only one housing condition, split evenly between the Standard housing (n = 3) and Complex housing (n = 3). The other half of tarantulas (n_female_ = 4; n_male_ = 2) entered both housing conditions during the test period. Tarantulas who entered both housing conditions, on average, entered the Standard housing 3.17 (SD = 1.07) times and the Complex housing 2.00 (SD = 1.40) times per 24 hr trial. The mean proportion of time spent between the two housing conditions was also approximately equal for these individuals (M_standard_ = 0.55, SD = 0.44; M_complex_ = 0.450, SD = 0.44; M_difference_ = 0.10, CI: -0.68, 0.88). Housing condition prior to preference testing did not appear to affect tarantula preferences as the housing condition prior to testing was the first choice for only 50% of the tarantulas.

## Discussion

### Do species-typical behavior, space use patterns, and/or physical attributes differ between housing conditions?

In both the Standard and Complex conditions, the tarantulas spent nearly all their time inactive suggesting housing had a limited effect on their activity budgets. Though there is little research on this species in nature, the family Theraphosidae has generally been described as sedentary, ambush predators [60]; however, males may be more active than females during seasonal breeding periods [61]. Thus, it stands to reason that the tarantulas may not adjust their activity patterns based on exhibit complexity the way more active taxa do. Alternatively, the tarantulas may choose to be more active in other housing designs not evaluated here. Future studies should consider a broader range of designs than this preliminary study. To expand on this point, our Complex habitat increased size and physical complexity simultaneously. Controlling for one or the other in future studies will additionally help identify what tarantulas benefit from the most.

We hypothesized that the increased structural elements provided by the Complex housing may provide more opportunity for engagement in species-typical behavior, such as web construction, but this was not observed. Few instances of web construction were recorded during behavioral observations, though webbing was present in many of the tarantulas’ spaces throughout the study. Thus, this behavior was occurring, and our sampling procedure was inadequate at quantifying differences between conditions.

Tarantulas used more space in general (home ranges) and spent most of their time within a larger space (core areas) in the Complex housing compared to the Standard housing. It is often thought that arachnids do not have significant space requirements for proper care [58]; however, our study found that tarantulas will utilize more space if it is provided to them, suggesting their space requirements are likely greater than generally perceived. In either housing condition, the tarantulas did not utilize the entire surface area available during observations, though that likely does not suggest either space was “too much” for them as studies in other species have shown having the choice to use or not use space is an important component of animal care and welfare [10, 80].

For animals in human care, the quality of space provided is just as, if not more, important than the quantity of space provided [1]. In the Complex housing condition, the tarantulas were given several enclosure elements that varied in size, shape, and extent of cover. The tarantulas did not spend time on top of these elements often and no meaningful differences in use were observed between housing conditions. Thus, a focus on climbing structures may not be a high priority when designing enclosures for these tarantulas, which is unsurprising for a terrestrial species. The Standard housing condition contained a piece of cork bark, which could be used structurally as cover if the tarantulas were to dig around it, but this was not observed. When offered hides that did not require digging in the Complex condition the tarantulas utilized them. Therefore, the availability of readily usable hides may be an important element for Brazilian black tarantula husbandry.

On average, the tarantulas utilized the branch and log zones of the Complex housing condition more than expected based on availability, indicating a preference for these zones. In situ, Brazilian black tarantulas have been found in rocky, open field areas [59] and may be found underneath rocks, fallen trunks, logs, other natural crevices, or dug burrows [60]. With the exception of males searching for females, they are thought to spend most of their time inside or near their burrows [60]. Thus, we suggest that spending concentrated times in and at the entrance of these preferred areas is a species-typical behavior for Brazilian black tarantulas, elicited by the hide elements and nearby open spaces provided in the Complex housing condition. These findings provide additional evidence that offering hides may be an important aspect of tarantula care and welfare. In terms of the enclosure elements rarely used by the tarantulas it is important to note that the absence of active use does not necessarily indicate a lack of benefit provided by these elements [80].

Tarantulas in Standard housing spent nearly fivefold more time on the wall of the enclosure than when in the Complex housing. Given these tarantula’s terrestrial nature [58], it is surprising such a notable difference in use of vertical space was observed between conditions. More so this difference is surprising because the amount of available wall space in the Complex housing was nearly 4 times greater than the Standard housing. This suggests an aspect of the Standard housing was eliciting this behavior that was absent in the Complex housing. In reptiles, ITB is a stereotypic behavior associated with enclosures that are limited in complexity and is hypothesized to be associated with escape or a desire to find cover [35, 36]. While time spent on the wall and reptile ITB are not the same expression of behavior, it could be that time spent on the wall for tarantulas serves a similar function as ITB in reptiles. It is also worth noting that falls only occurred when tarantulas were on walls. This suggests that the odds of an injury from a fall are more likely in the Standard housing where tarantula spent more time on the walls than in the Complex housing.

### Do responses to novelty differ between housing conditions?

During the novel environment test, tarantulas were more active following the Complex housing condition compared to the Standard housing condition. Cognitive bias tests, such as this novel environment test, attempt to gain insight into the animal’s affective state [47]. Studies of other species have similarly found that increasing environmental complexity alters behavioral and neural responses during these tests [19, 22, 27, 53, 55, 81]. The findings here suggest that following the Complex housing condition, tarantula behavior was more neophilic and exploratory, which in other contexts have been described as more optimistic emotional states [27, 47, 48, 55]. Other studies have similarly identified increased neophilia in other arachnid species following time spent in a complex living environment compared to a simpler environment [32, 56, 57], including a study of tarantulas in the same Theraphosidae family. If the tarantulas studied here are in fact more neophilic because of the Complex housing condition, then this would likely benefit the tarantulas’ overall welfare in human care, as they may cope better with the sometimes stressful and often dynamic nature of an ex-situ environment. However, interpreting this difference in tarantula behavior is speculative, as differing species may react differently given their specific behavioral repertoire [37] and because so little has been studied on Brazilian black tarantulas. Thus, we state these considerations cautiously.

### Do tarantulas exhibit a preference for either housing condition?

The tarantulas did not demonstrate a preference for either enclosure when presented with both simultaneously. Given their overall inactivity, it may be unsurprising that about half of the tarantulas selected a side and explored no further. It may be that the study period was insufficient to gauge exhibit preferences in this species. An improved study design for future inquiries would likely benefit from an extended timeframe within the preference test apparatus. However, half of the tarantulas did explore both sides before making a selection. Even those that explored both sides showed no preference for one enclosure over the other, on average. This is more difficult to interpret and suggests this testing apparatus was either not testing preferences or that the tarantulas truly had no preference between the two enclosures. Preference testing is a valuable approach in understanding non-human animal behavior and needs, however, methodologies need to be appropriate for the species being tested [8, 16, 24, 26]. We likely have room to grow in improving our understanding of how to design and employ preference testing for tarantulas. As mentioned, increasing study duration may improve the functionality of this approach. Additionally, providing variation in complexity within one larger housing space, rather than requiring individuals to travel between enclosures, may be better suited for tarantula preference testing.

## Conclusion

The behavior, and ultimately welfare, of invertebrates living in human care is poorly studied, leaving a gap in evidence-based research needed to provide guidance on their optimal management. This study found that tarantulas were primarily inactive. Activity budget data alone may make interpreting welfare statuses difficult, creating challenges for the care of this commonly managed taxa. Here, in addition to traditional activity budget monitoring, we assessed patterns of space use and used two experimental paradigms to better understand the behavior of tarantulas in relation to environmental complexity. Like other taxa, the tarantulas utilized more space when it was provided and were more neophilic when housed in a more complex environment. This suggests that despite the tarantulas being predominately inactive in both environments and displaying no clear preference for either environment, the Complex housing condition may have had positive effects on their overall welfare status. While preliminary and in need of replication with larger sample sizes, this study provides insights into the behavior and welfare of tarantulas living in human care and how future studies of invertebrate behavior and welfare can be designed.

## Supporting information

S1 File Data deposit. This Microsoft Excel Workbook contains the original data analyzed in the main text, including activity and space use scan data, all occurrence data, location data, and data from the novel environment and preference tests.
(XLSX)

S2 File Supplementary tables. (DOCX)

S3 File Supplementary figures. (DOCX)
