# Correction: Tarantula welfare may be improved with greater environmental complexity: A preliminary behavioral study with Brazilian black tarantulas (*Grammastola pulchra*)

**DOI:** 10.1371/journal.pone.0321123

**Published:** 2025-03-25

**Authors:** Laura Stalter, Tayler Dorleus, Nicholas Milone, Jamie Sincage, Michelle Skurski, Austin Leeds

The genus name “*Grammostola*” is misspelled in the article title. The correct title is: Tarantula welfare may be improved with greater environmental complexity: A preliminary behavioral study with Brazilian black tarantulas (*Grammostola pulchra*). The correct citation is: Stalter L, Dorleus T, Milone N, Sincage J, Skurski M, Leeds A (2024) Tarantula welfare may be improved with greater environmental complexity: A preliminary behavioral study with Brazilian black tarantulas (*Grammostola pulchra*). PLoS ONE 19(12): e0314501. https://doi.org/10.1371/journal.pone.0314501.

In the Abstract, there is an error in the second sentence. The correct sentence is: Here we conducted a preliminary investigation of Brazilian black tarantula (*Grammostola pulchra*) behavior in relation to environmental complexity.

In the Introduction, there is an error in the second sentence of the fifth paragraph. The correct sentence is: At Disney’s Animal Kingdom®, Brazilian black tarantulas (*Grammostola pulchra*) are managed in an off-exhibit building and housed in containers 27x27x21 cm in size with substrate 2.5 cm deep, furnished with a single piece of cork bark and a water dish.
