# Supplementary material for: Tarantula welfare may be improved with greater environmental complexity: A preliminary behavioral study with Brazilian black tarantulas (Grammastola pulchra)
Source: PLoS One. 2024 Dec 5;19(12):e0314501. doi: 10.1371/journal.pone.0314501 (PMC11620463; doi:10.1371/journal.pone.0314501)
Supplement: S2 File — (DOCX) [file pone.0314501.s002.docx]

**Supplementary Tables**

**S1 Table.** Description of subject removal per study condition. Check mark denotes data are included in analysis. X denotes data are not included in analysis or missing. Tarantulas were removed if shedding occurred because the energetic costs can alter species-typical behavior.

| **Tarantula** | **Sex** | **Study Condition** | | |
| --- | --- | --- | --- | --- |
|  |  | **Comparing Housing Condition**  **(Standard and Complex)** | **Novel Environment Test**  **(Post Each Housing Condition)** | **Preference Test** |
| 120154 | F | X  Removed from analysis due to being not visible for entirety of Complex condition. | ✓ | X  Did not participate due to shedding. |
| 120155 | F | ✓ | ✓ | ✓ |
| 120156 | M | ✓ | ✓ | ✓ |
| 120157 | F | ✓ | ✓ | ✓ |
| 120158 | F | ✓ | X  Video recording error. | ✓ |
| 120159 | F | ✓ | ✓ | ✓ |
| 120160 | F | ✓ | ✓ | ✓ |
| 120161 | F | ✓ | X  Video recording error. | ✓ |
| 120162 | F | ✓ | ✓ | ✓ |
| 120163 | M | ✓ | X  Video recording error. | ✓ |
| 120165 | M | ✓ | X  Video recording error. | ✓ |
| 120168 | F | ✓ | X  Video recording error. | ✓ |
| 130847 | M | X  Removed from study due to shedding in first housing condition. | X  Removed from study due to shedding in first housing condition. | X  Removed from study due to shedding in first housing condition. |
| 130850 | F | ✓ | ✓ | ✓ |
| 130851 | F | ✓ | ✓ | ✓ |

**S2 Table**. Ethogram defining use of enclosure elements and vertical space use.

| **Enclosure Element Use** |  |
| --- | --- |
| In Hide | Tarantula is under provided hide. For Complex condition, modifier notes which hide is occupied. Hide may be a plant if >50% of the tarantula’s body is covered by a leaf. |
| Furniture | Tarantula has >50% of body on enclosure element, including water dish. Modifier notes which item. |
| **Vertical Space Use** |  |
| Underground | Tarantula has >50% of body below the surface of the substrate. If 50/50 split, tarantula’s location favors where pedipalps are. |
| Ground | Tarantula has >50% of body on the bottom of the enclosure, not including furniture items. If 50/50 split, tarantula’s location favors where pedipalps are. |
| Furniture | Tarantula has >50% of body on enclosure element, including water dish. Modifier notes which item. If 50/50 split, tarantula’s location favors where pedipalps are. |
| Wall | Tarantula has >50% of body on the enclosure wall. If 50/50 split, tarantula’s location favors where pedipalps are. |
| Ceiling | Tarantula has >50% of body on the enclosure lid. If 50/50 split, tarantula’s location favors where pedipalps are. |

**S3 Table.** Male tarantula activity budget by housing condition and time of day. Values rounded to three decimal places, anything below three decimal places are rounded to 0.000. Unobserved behaviors are noted as such.

| **Behavior** | **Mean Proportion of Time** | | | | | | |
| --- | --- | --- | --- | --- | --- | --- | --- |
|  | **Dark Hours** | | | **Light Hours** | | | |
|  | **Standard Housing (SD)** | **Complex Housing (SD)** | **Mean Difference (95% CI)** | **Standard Housing (SD)** | | **Complex Housing (SD)** | **Mean Difference (95% CI)** |
| Inactive | 0.926 (0.066) | 0.965  (0.033) | -0.039  (-0.078, -0.001) | 0.964 (0.032) | 0.959 (0.049) | | 0.005  (-0.043, 0.054) |
| Locomotion | 0.009 (0.010) | 0.011  (0.008) | -0.001  (-0.008, 0.005) | 0.006 (0.004) | 0.011 (0.019) | | -0.006  (-0.023, 0.012) |
| Climb | 0.022 (0.029) | 0.010  (0.015) | 0.012  (-0.002, 0.027) | 0.003 (0.006) | Unobserved | | Not Applicable |
| Active in Place | 0.029 (0.027) | 0.013  (0.013) | 0.016  (-0.005, 0.038) | 0.007 (0.005) | 0.029 (0.051) | | -0.022  (-0.075, 0.030) |
| Preen | 0.009 (0.006) | Unobserved | Not Applicable | 0.001 (0.001) | 0.001 (0.001) | | 0.000  (-0.002, 0.002) |
| Web Construction | 0.005 (0.009) | 0.002  (0.003) | 0.003  (-0.006, 0.013) | 0.016 (0.025) | Unobserved | | Not Applicable |
| Dig/Burrow | Unobserved | Unobserved | Not Applicable | 0.003 (0.006) | Unobserved | | Not Applicable |
| Leg Raise | Unobserved | Unobserved | Not Applicable | Unobserved | Unobserved | | Not Applicable |
| Other | Unobserved | Unobserved | Not Applicable | Unobserved | Unobserved | | Not Applicable |

**S4 Table.** Female tarantula activity budget by housing condition and time of day. Values rounded to three decimal places, anything below three decimal places are rounded to 0.000. Unobserved behaviors are noted as such.

| **Behavior** | **Mean Proportion of Time** | | | | | |
| --- | --- | --- | --- | --- | --- | --- |
|  | **Dark Hours** | | | **Light Hours** | | |
|  | **Standard Housing (SD)** | **Complex Housing (SD)** | **Mean Difference (95% CI)** | **Standard Housing (SD)** | **Complex Housing (SD)** | **Mean Difference (95% CI)** |
| Inactive | 0.966  (0.028) | 0.973  (0.026) | -0.008  (-0.025, 0.009) | 0.961 (0.032) | 0.972 (0.028) | -0.002  (-0.024, 0.020) |
| Locomotion | 0.008 (0.008) | 0.008 (0.009) | 0.000  (-0.004, 0.005) | 0.004 (0.003) | 0.004 (0.006) | -0.001  (-0.005, 0.003) |
| Climb | 0.014 (0.014) | 0.008 (0.009) | 0.007  (-0.001, 0.015) | 0.014 (0.011) | 0.007 (0.010) | 0.005  (-0.002, 0.012) |
| Active in Place | 0.008 (0.009) | 0.008 (0.012) | 0.000  (-0.008, 0.009) | 0.017 (0.020) | 0.014 (0.016) | -0.002  (-0.015, 0.010) |
| Preen | 0.002 (0.005) | 0.003 (0.010) | -0.002  (-0.009, 0.005) | 0.001 (0.002) | 0.002 (0.004) | -0.001  (-0.004, 0.002) |
| Web Construction | 0.002 (0.004) | Unobserved | Not Applicable | 0.003 (0.006) | 0.001 (0.004) | 0.000  (-0.004, 0.006) |
| Dig/Burrow | Unobserved | Unobserved | Not Applicable | 0.001 (0.002) | Unobserved | Not Applicable |
| Leg Raise | Unobserved | Unobserved | Not Applicable | Unobserved | Unobserved | Not Applicable |
| Other | 0.000 (0.000) | Unobserved | Not Applicable | 0.000 (0.001) | Unobserved | Not Applicable |

**S5 Table.** Generalized linear mixed model outputs for predictors of home range size. Parameter estimates with ‶-″ are compared to remaining variable conditions within each predictor variable.

| **Predictor Variable** | **χ^2^** | **D.F.** | **Variable Condition** | **Estimate** | **S.E.** | **z** |
| --- | --- | --- | --- | --- | --- | --- |
| Intercept | 43.104 | 1 | - | 281.354 | 42.854 | 6.565 |
| Housing | 6.814 | 1 | Complex | - | - | - |
|  |  |  | Standard | -123.046 | 47.139 | -2.610 |
| Sex | 0.002 | 1 | Female | - | - | - |
|  |  |  | Male | 3.051 | 66.527 | 0.046 |

**S6 Table.** Generalized linear mixed model outputs for predictors of core area size. Parameter estimates with ‶-″ are compared to remaining variable conditions within each predictor variable.

| **Predictor Variable** | **χ^2^** | **D.F.** | **Variable Condition** | **Estimate** | **S.E.** | **z** |
| --- | --- | --- | --- | --- | --- | --- |
| Intercept | 56.469 | 1 | - | 63.353 | 8.431 | 7.515 |
| Housing | 10.643 | 1 | Complex | - | - | - |
|  |  |  | Standard | -34.958 | 10.715 | -3.262 |
| Sex | 0.172 | 1 | Female | - | - | - |
|  |  |  | Male | 4.961 | 11.980 | 0.414 |

**S7 Table.** Generalized linear mixed model outputs for predictors of the proportion of scans on the wall at night. Parameter estimates with ‶-″ are compared to remaining variable conditions within each predictor variable.

| **Predictor Variable** | **χ^2^** | **D.F.** | **Variable Condition** | **Estimate** | **S.E.** | **z** |
| --- | --- | --- | --- | --- | --- | --- |
| Intercept | 87.366 | 1 | - | -3.940 | 0.422 | -9.347 |
| Housing | 17.430 | 1 | Complex | - | - | - |
|  |  |  | Standard | 1.602 | 0.384 | 4.175 |
| Sex | 0.380 | 1 | Female | - | - | - |
|  |  |  | Male | 0.301 | 0.489 | 0.616 |

**S8 Table.** Generalized linear mixed model outputs for predictors of the proportion of scans on the wall during the day. Parameter estimates with ‶-″ are compared to remaining variable conditions within each predictor variable.

| **Predictor Variable** | **χ^2^** | **D.F.** | **Variable Condition** | **Estimate** | **S.E.** | **z** |
| --- | --- | --- | --- | --- | --- | --- |
| Intercept | 53.948 | 1 | - | -3.130 | 0.426 | -7.345 |
| Housing | 14.419 | 1 | Complex | - | - | - |
|  |  |  | Standard | 1.416 | 0.373 | 3.797 |
| Sex | 1.199 | 1 | Female | - | - | - |
|  |  |  | Male | -00674 | 0.616 | -1.095 |

**S9 Table.** Generalized linear mixed model outputs for predictors of the proportion of feeds in which a tarantula did not eat. Parameter estimates with ‶-″ are compared to remaining variable conditions within each predictor variable.

| **Predictor Variable** | **χ^2^** | **D.F.** | **Variable Condition** | **Estimate** | **S.E.** | | **z** |
| --- | --- | --- | --- | --- | --- | --- | --- |
| Intercept | 5.344 | 1 | - | -3.344 | 1.447 | -2.312 | |
| Housing | 0.000 | 1 | Complex | - | - | - | |
|  |  |  | Standard | <-0.001 | 1.688 | 0.000 | |
| Sex | 0.406 | 1 | Female | - | - | - | |
|  |  |  | Male | 1.075 | 1.689 | 0.637 | |

**S10 Table.** Generalized linear mixed model outputs for predictors of weight. Parameter estimates with ‶-″ are compared to remaining variable conditions within each predictor variable.

| **Predictor Variable** | **χ^2^** | **D.F.** | **Variable Condition** | **Estimate** | **S.E.** | **z** |
| --- | --- | --- | --- | --- | --- | --- |
| Intercept | 272.357 | 1 | - | 19.621 | 1.189 | 16.503 |
| Housing | 0.047 | 1 | Complex | - | - | - |
|  |  |  | Standard | -0.033 | 0.153 | -0.217 |
| Sex | 0.974 | 1 | Female | - | - | - |
|  |  |  | Male | -2.267 | 2.298 | -0.987 |

**S11 Table.** Generalized linear mixed model outputs for predictors active behavior during the novel environment test. Parameter estimates with ‶-″ are compared to remaining variable conditions within each predictor variable.

| **Predictor Variable** | **χ^2^** | **D.F.** | **Variable Condition** | **Estimate** | **S.E.** | **z** |
| --- | --- | --- | --- | --- | --- | --- |
| Intercept | 18.577 | 1 | - | -1.362 | 0.316 | -4.310 |
| Housing | 17.732 | 1 | Complex | - | - | - |
|  |  |  | Standard | -0.918 | 0.218 | 4.211 |
| Sex | 2.175 | 1 | Female | - | - | - |
|  |  |  | Male | -1.072 | 0.727 | -1.475 |
