# Supplementary material for: Tarantula welfare may be improved with greater environmental complexity: A preliminary behavioral study with Brazilian black tarantulas (Grammastola pulchra)
Source: PLoS One. 2024 Dec 5;19(12):e0314501. doi: 10.1371/journal.pone.0314501 (PMC11620463; doi:10.1371/journal.pone.0314501)
Supplement: S3 File — (DOCX) [file pone.0314501.s003.docx]

**Supplementary Figures**


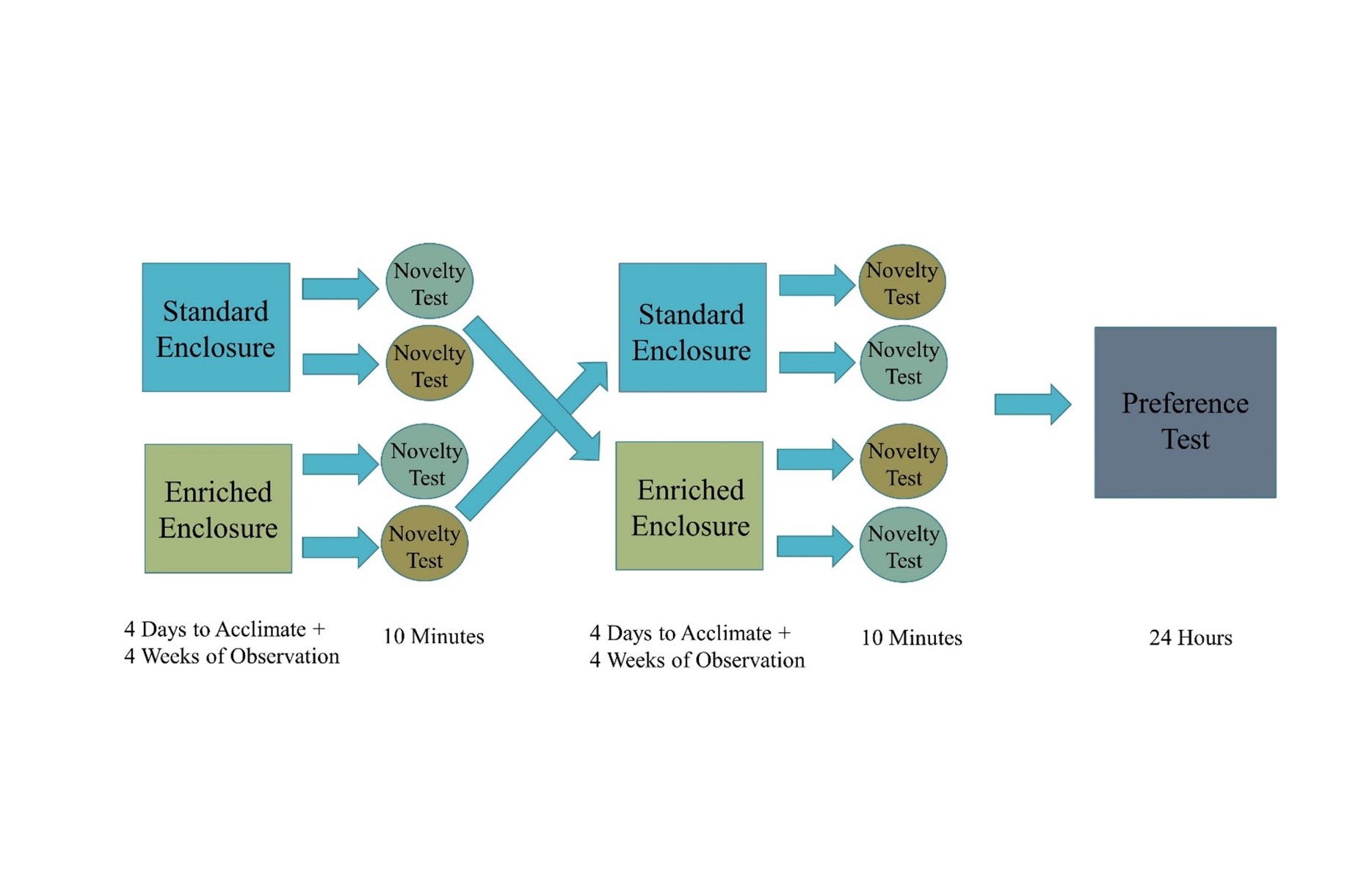
**S1 Figure.** Diagram of the experimental setup.


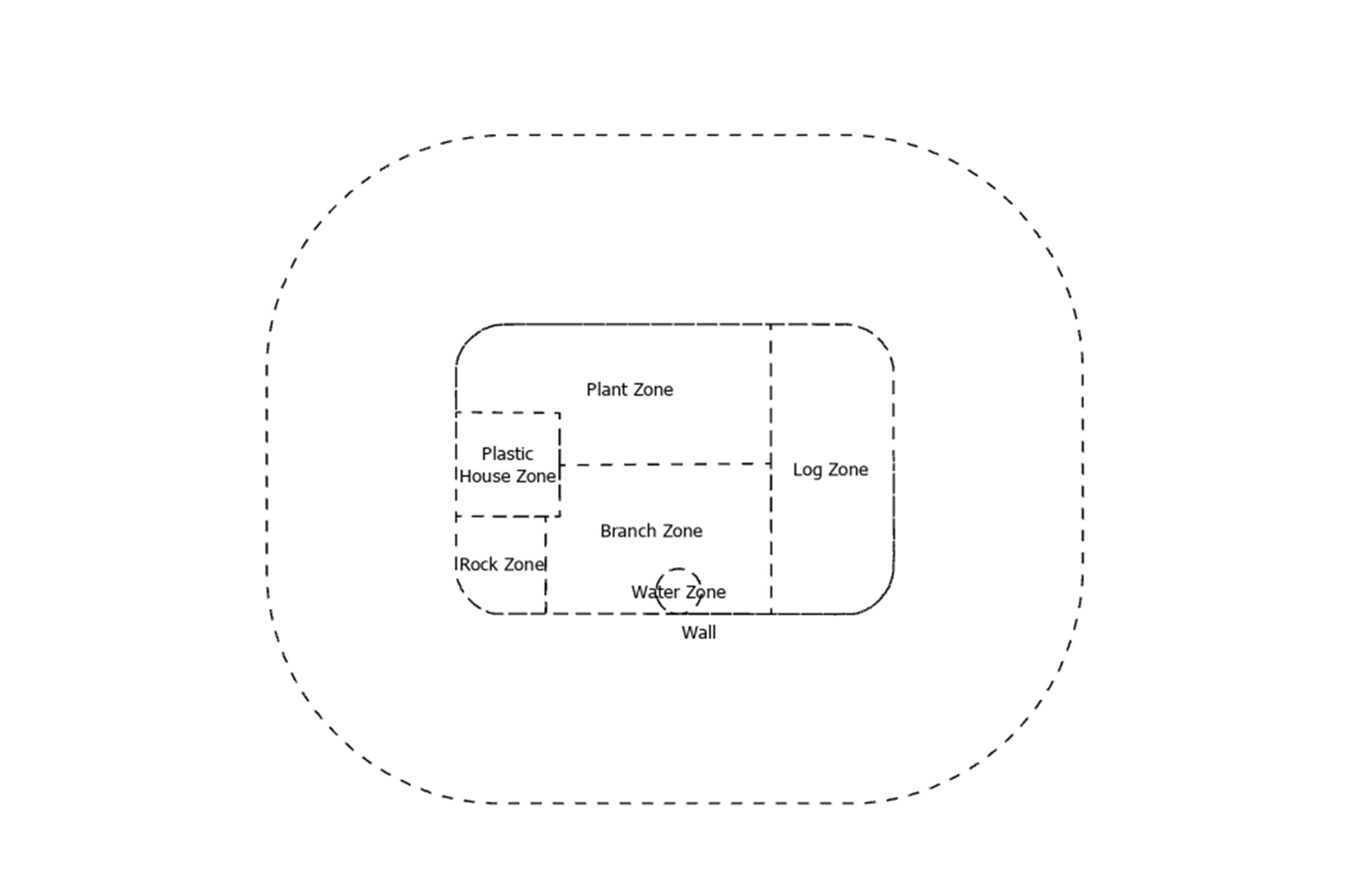
**S2 Figure.** Zones of the Complex housing condition.


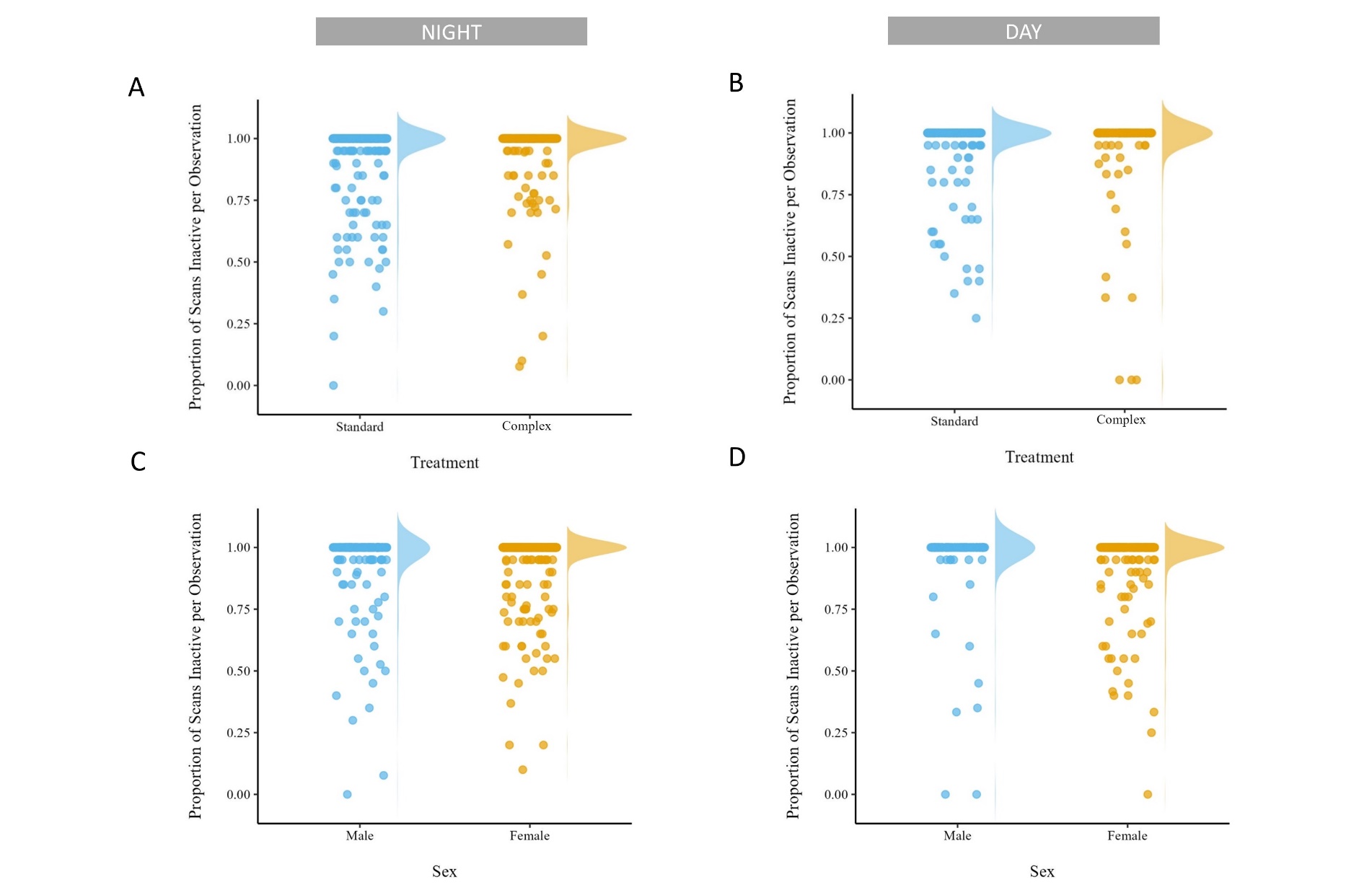
**S3 Figure.** Proportion of inactive behavior per observation by housing condition (A&B) and by sex (C&D) at night (left) and during the day (right) displayed via dot plot and distribution curve for all observations (n_night_ = 739, n_day_ = 487).


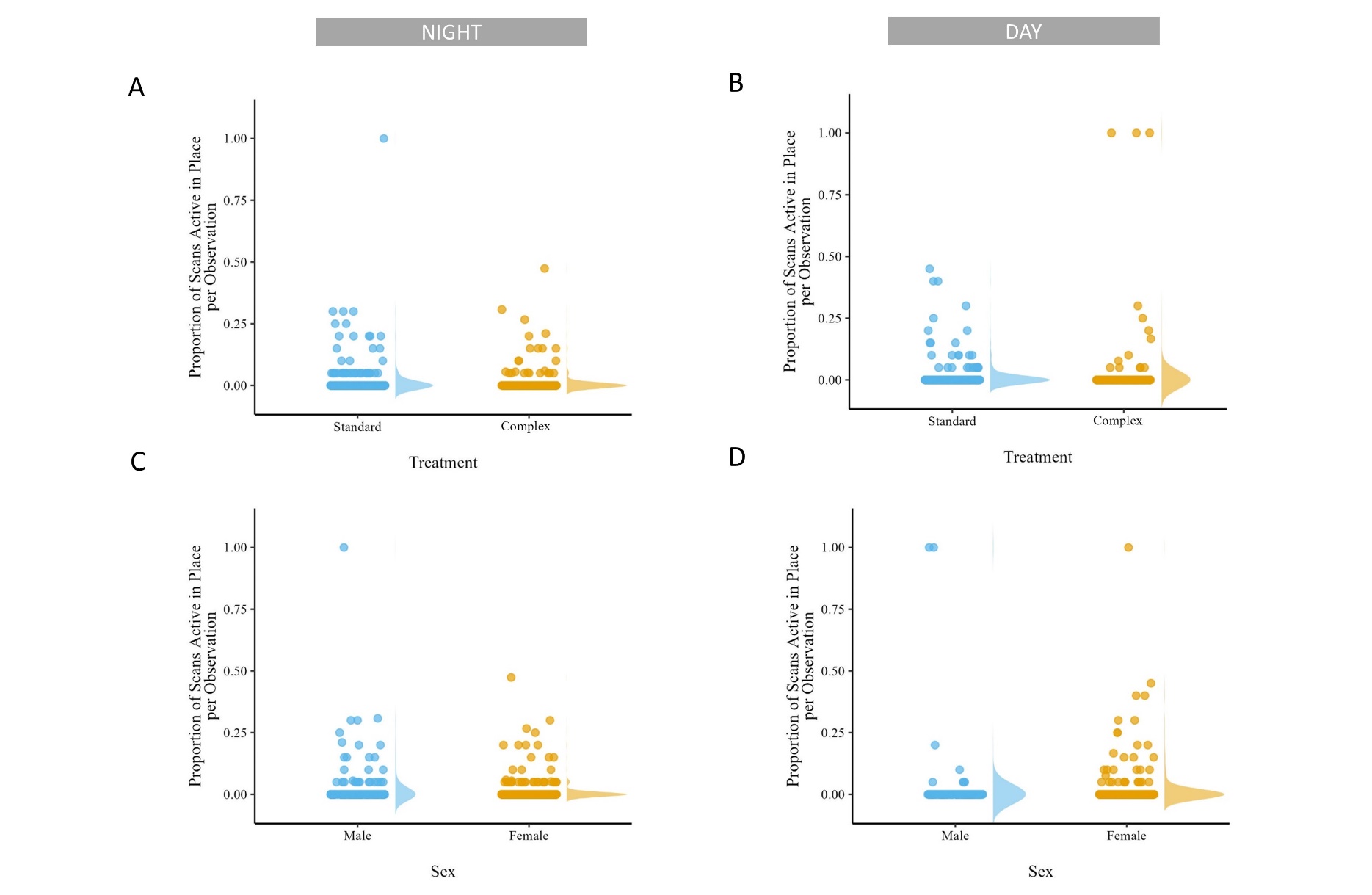
**S4 Figure.** Proportion of active in place behavior per observation by housing condition (A&B) and by sex (C&D) at night (left) and during the day (right) displayed via dot plot and distribution curve for all observations (n_night_ = 739, n_day_ = 487).


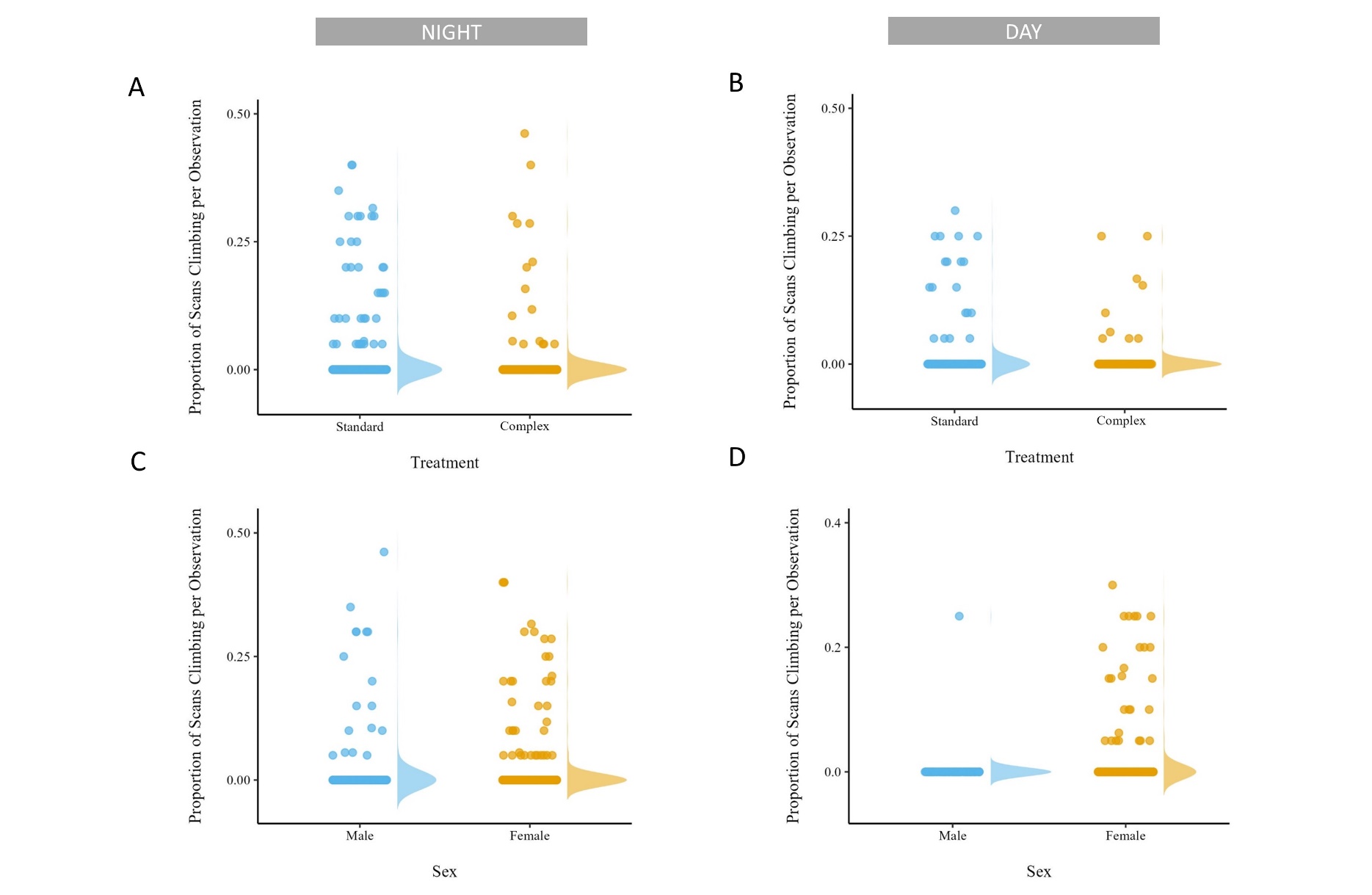
**S5 Figure.** Proportion of climbing behavior per observation by housing condition (A&B) and by sex (C&D) at night (left) and during the day (right) displayed via dot plot and distribution curve for all observations (n_night_ = 739, n_day_ = 487).


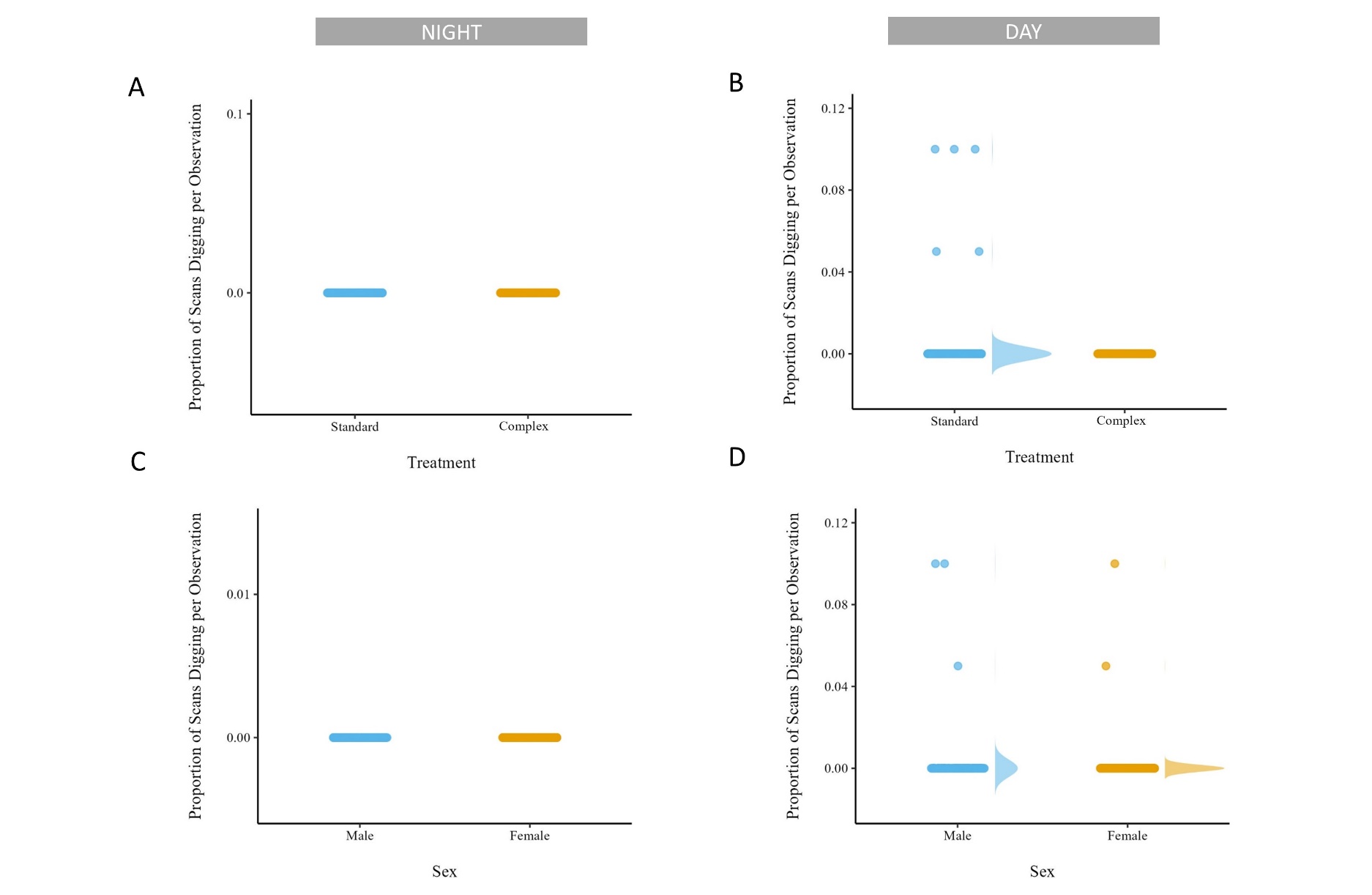
**S6 Figure.** Proportion of digging behavior per observation by housing condition (A&B) and by sex (C&D) at night (left) and during the day (right) displayed via dot plot and distribution curve for all observations (n_night_ = 739, n_day_ = 487).


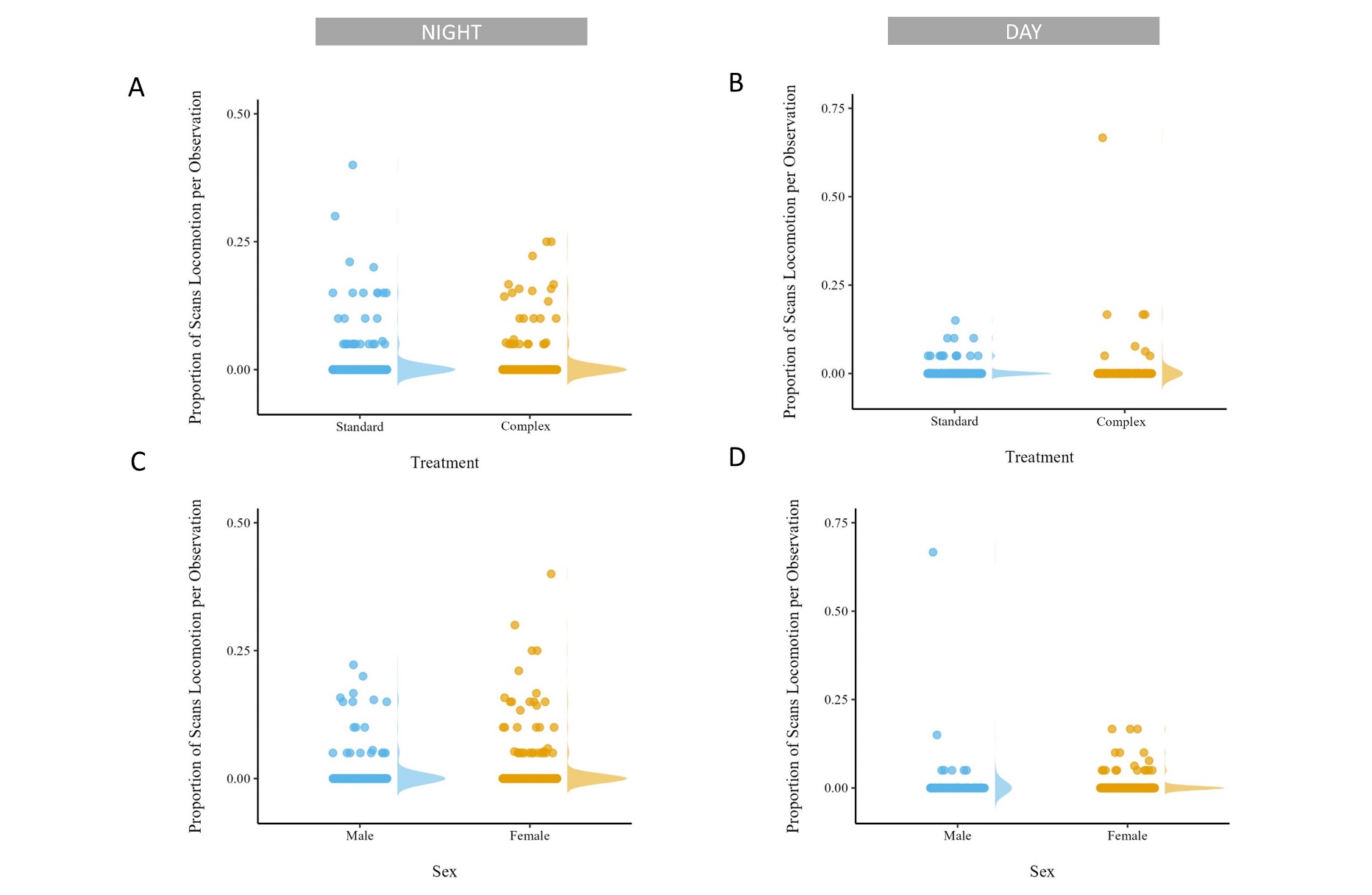
**S7 Figure.** Proportion of locomotion behavior per observation by housing condition (A&B) and by sex (C&D) at night (left) and during the day (right) displayed via dot plot and distribution curve for all observations (n_night_ = 739, n_day_ = 487).


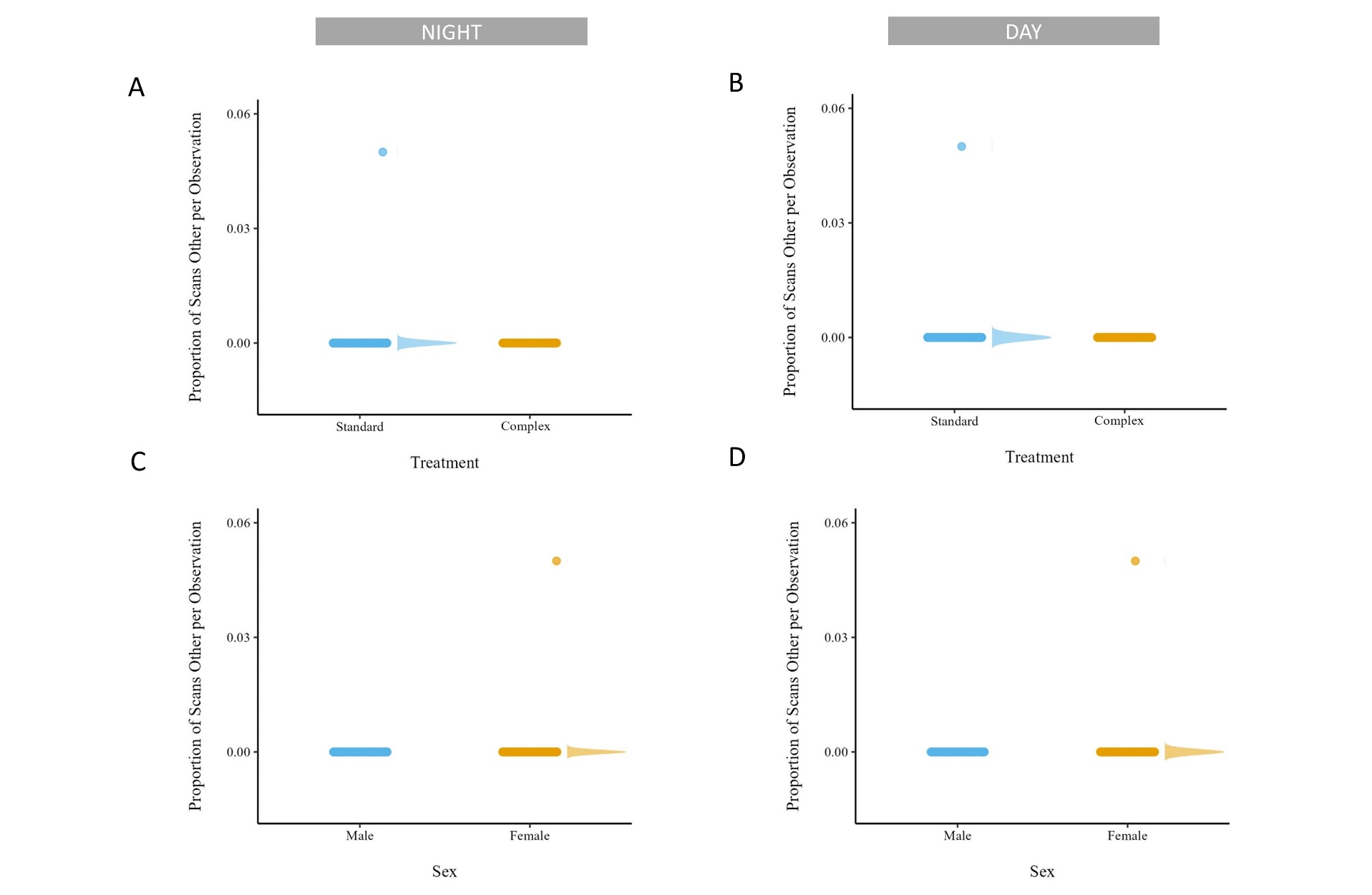
**S8 Figure.** Proportion of other behavior per observation by housing condition (A&B) and by sex (C&D) at night (left) and during the day (right) displayed via dot plot and distribution curve for all observations (n_night_ = 739, n_day_ = 487).


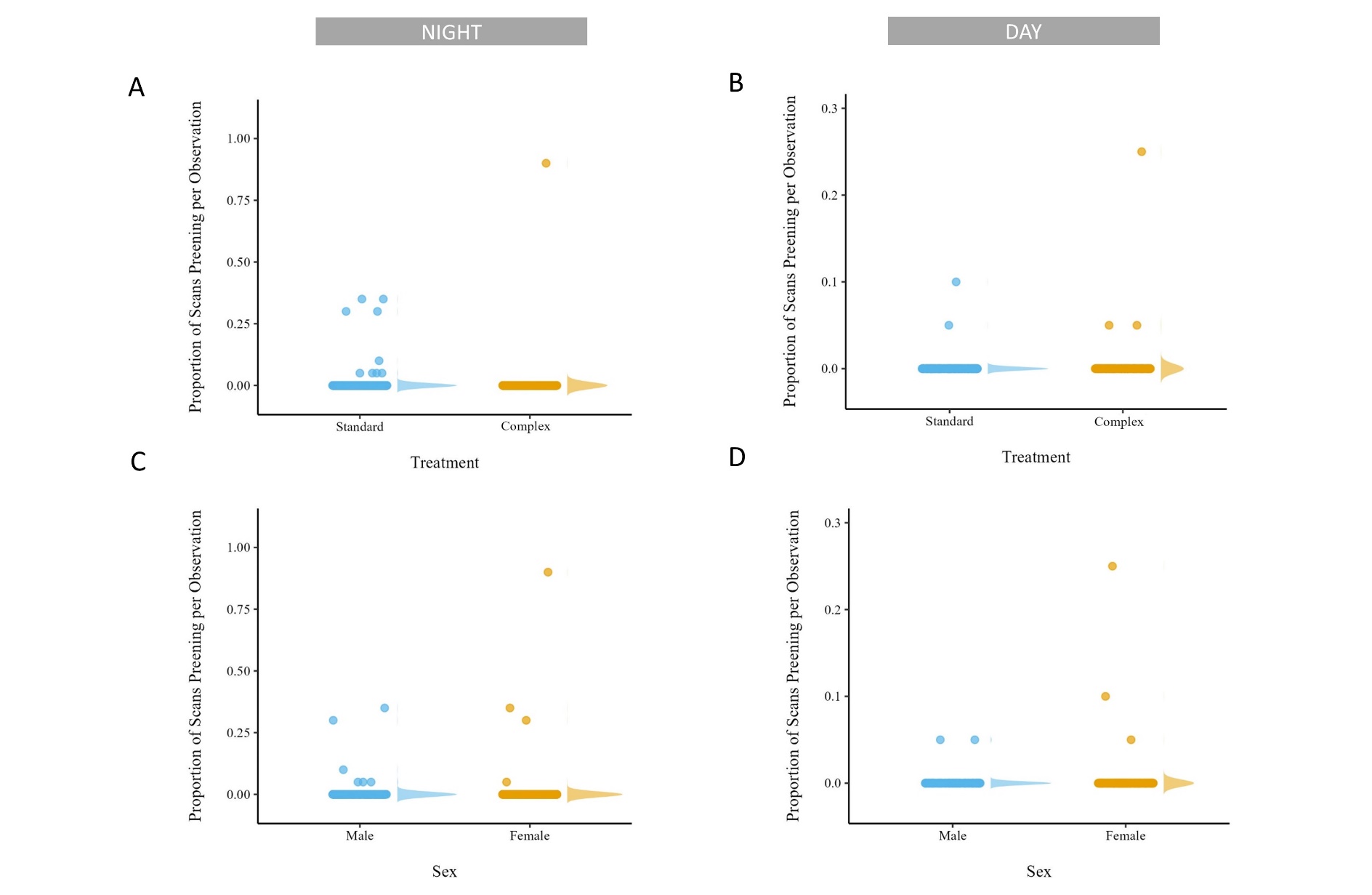
**S9 Figure.** Proportion of preening behavior per observation by housing condition (A&B) and by sex (C&D) at night (left) and during the day (right) displayed via dot plot and distribution curve for all observations (n_night_ = 739, n_day_ = 487).


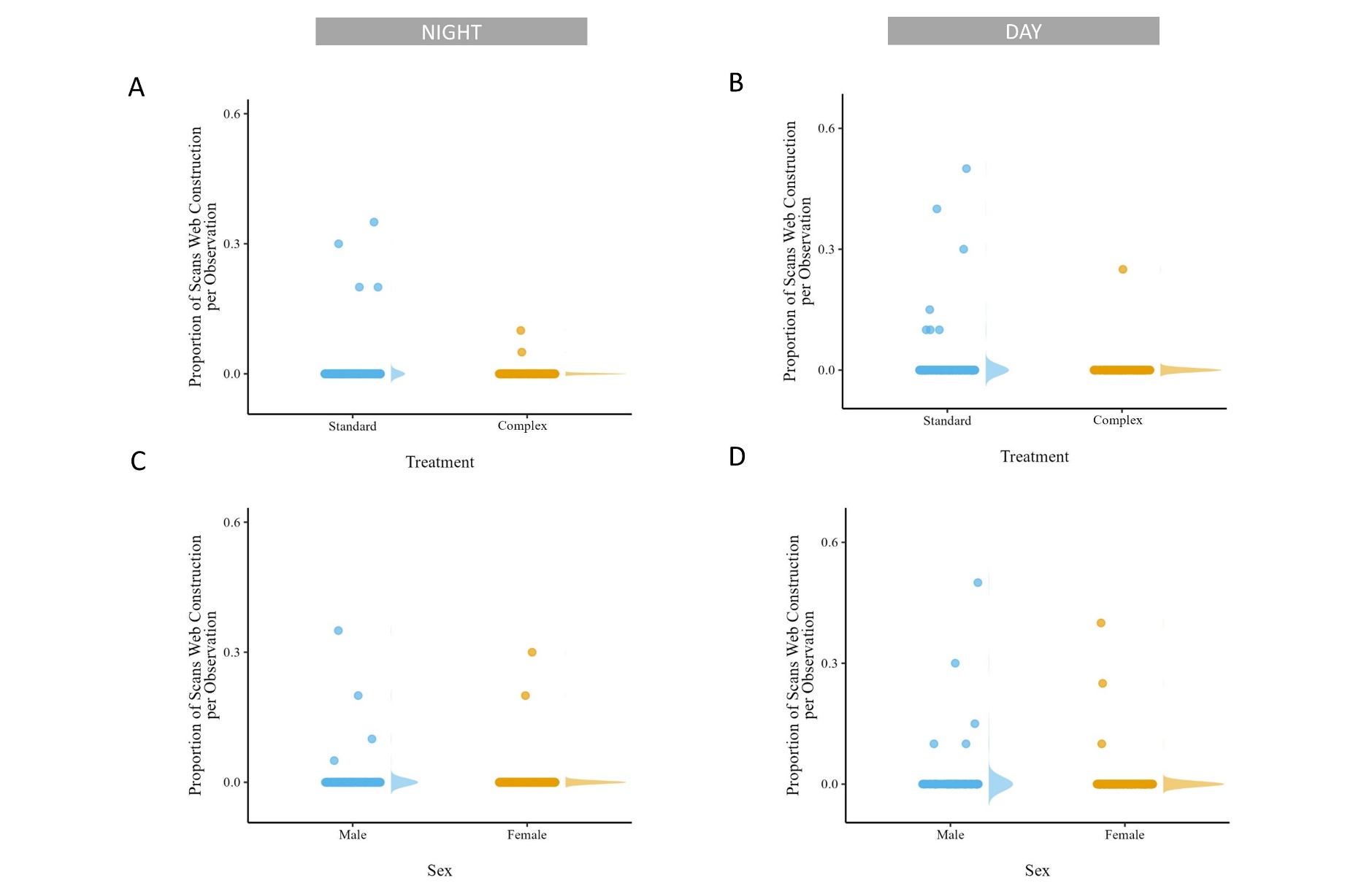
**S10 Figure.** Proportion of web construction behavior per observation by housing condition (A&B) and by sex (C&D) at night (left) and during the day (right) displayed via dot plot and distribution curve for all observations (n_night_ = 739, n_day_ = 487).


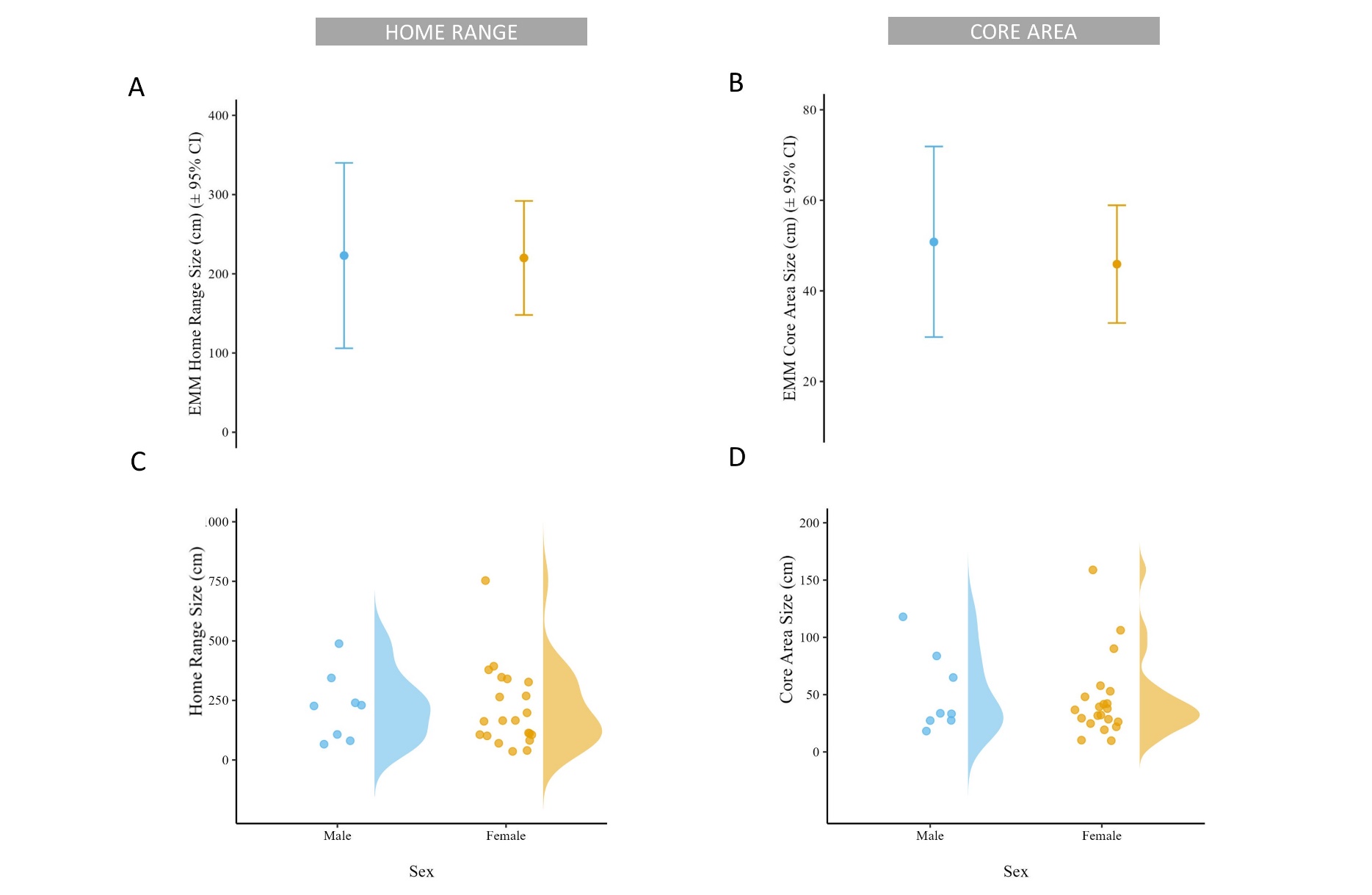
**S11 Figure.** A & B: estimated marginal mean (EMM) of home range size (A) and core area size (B) by sex with 95% confidence intervals. C & D: individual home range sizes (A) and core area sizes (B) by sex displayed via dot plot and distribution curve for all observations (n = 29).


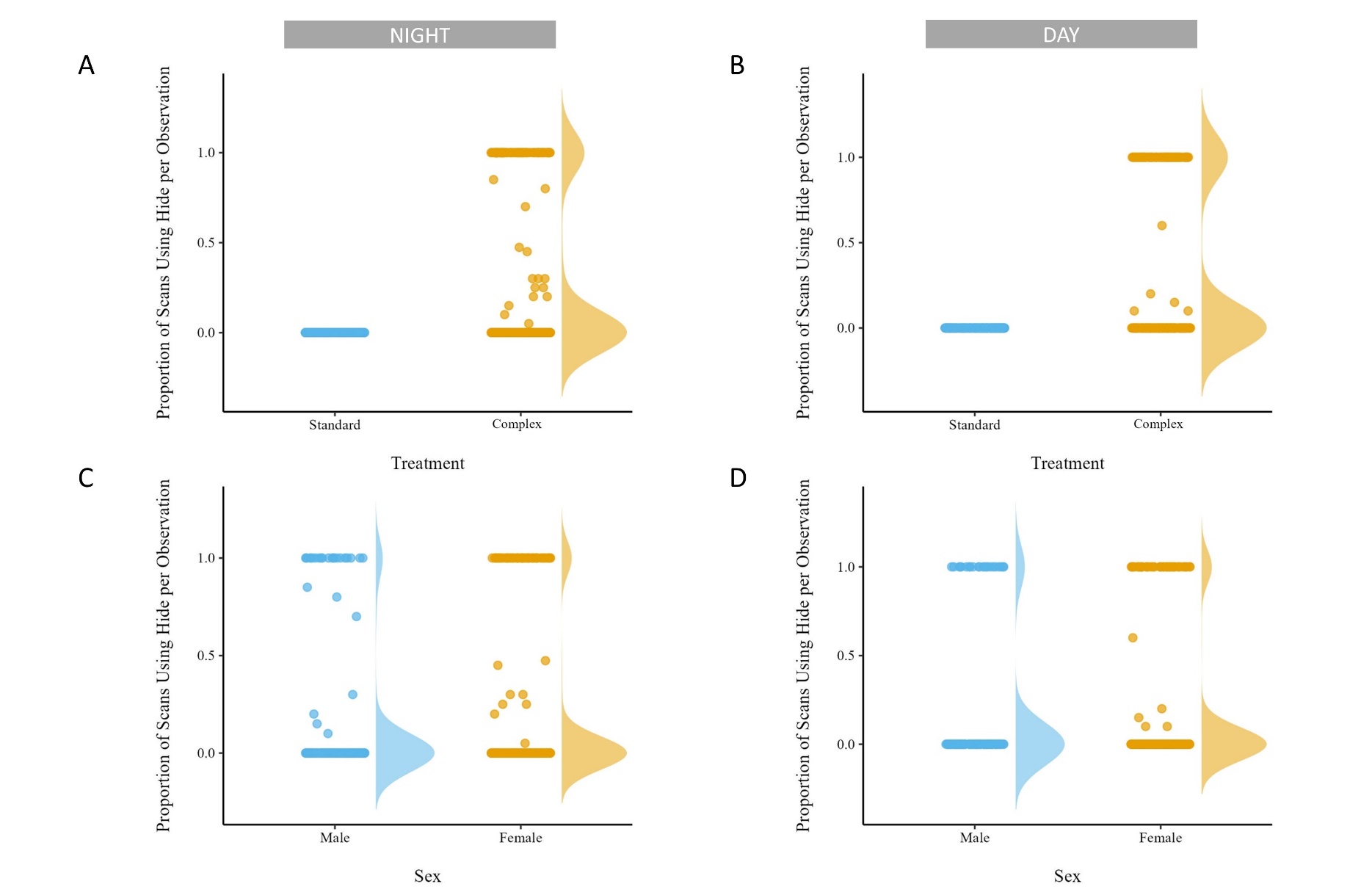
**S12 Figure.** A & B: proportion of hide use behavior per observation by housing condition at night (A) and during the day (B) displayed via dot plot and distribution curve for all observations (n_night_ = 789, n_day_ = 526). C & D: proportion of hide use behavior per observation by sex at night (C) and during the day (D) displayed via dot plot and distribution curve for all observations (n_night_ = 789, n_day_ = 526).


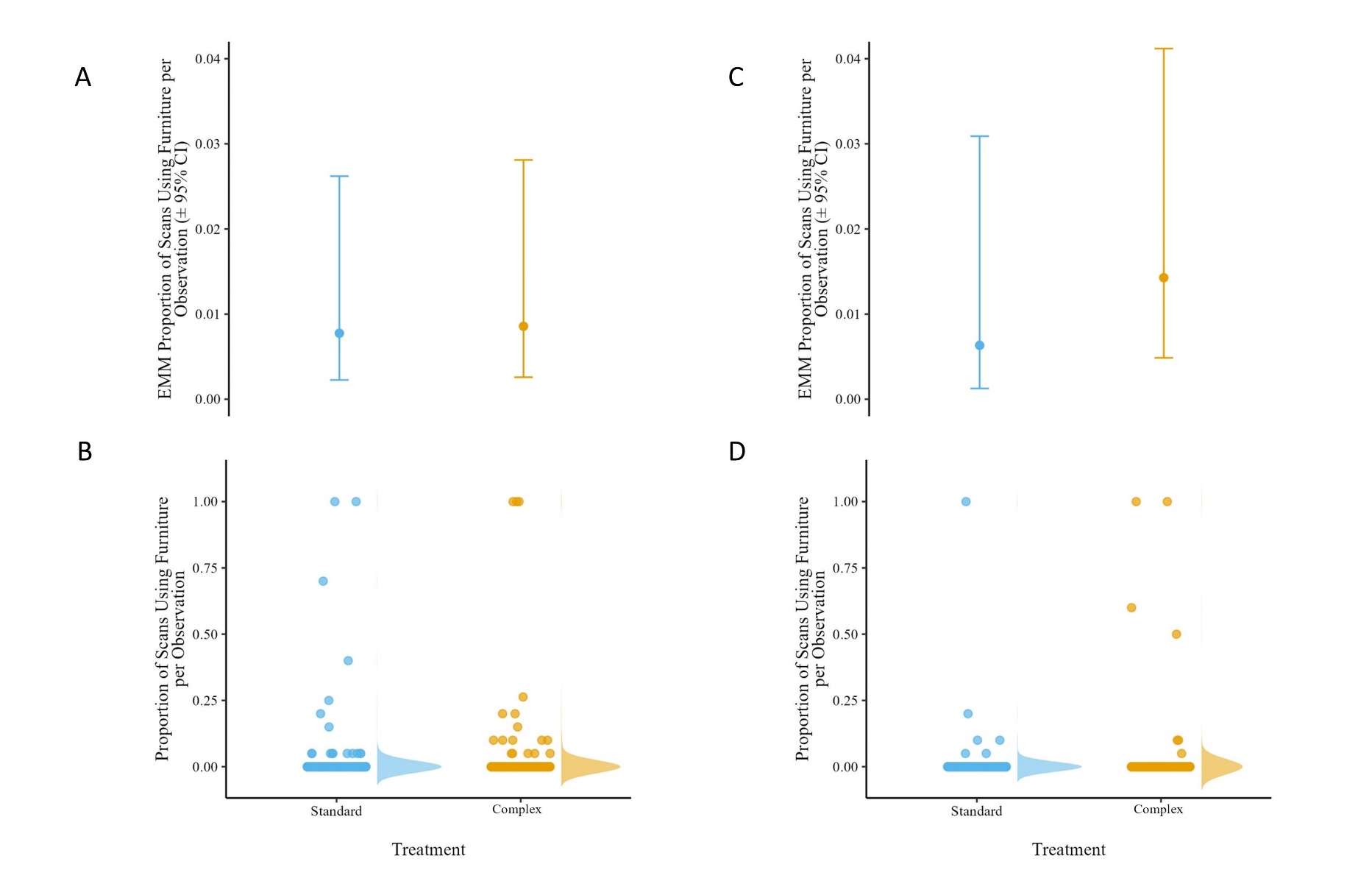
**S13 Figure.** A&C: estimated marginal mean (EMM) of proportion of furniture use behavior per observation by housing condition bounded by 95% confidence intervals (CI) at night (A) and during the day (C). B&D: proportion of furniture use behavior per observation by housing condition at night (B) and during the day (D) displayed via dot plot and distribution curve for all observations (n_night_ = 791, n_day_ = 531).


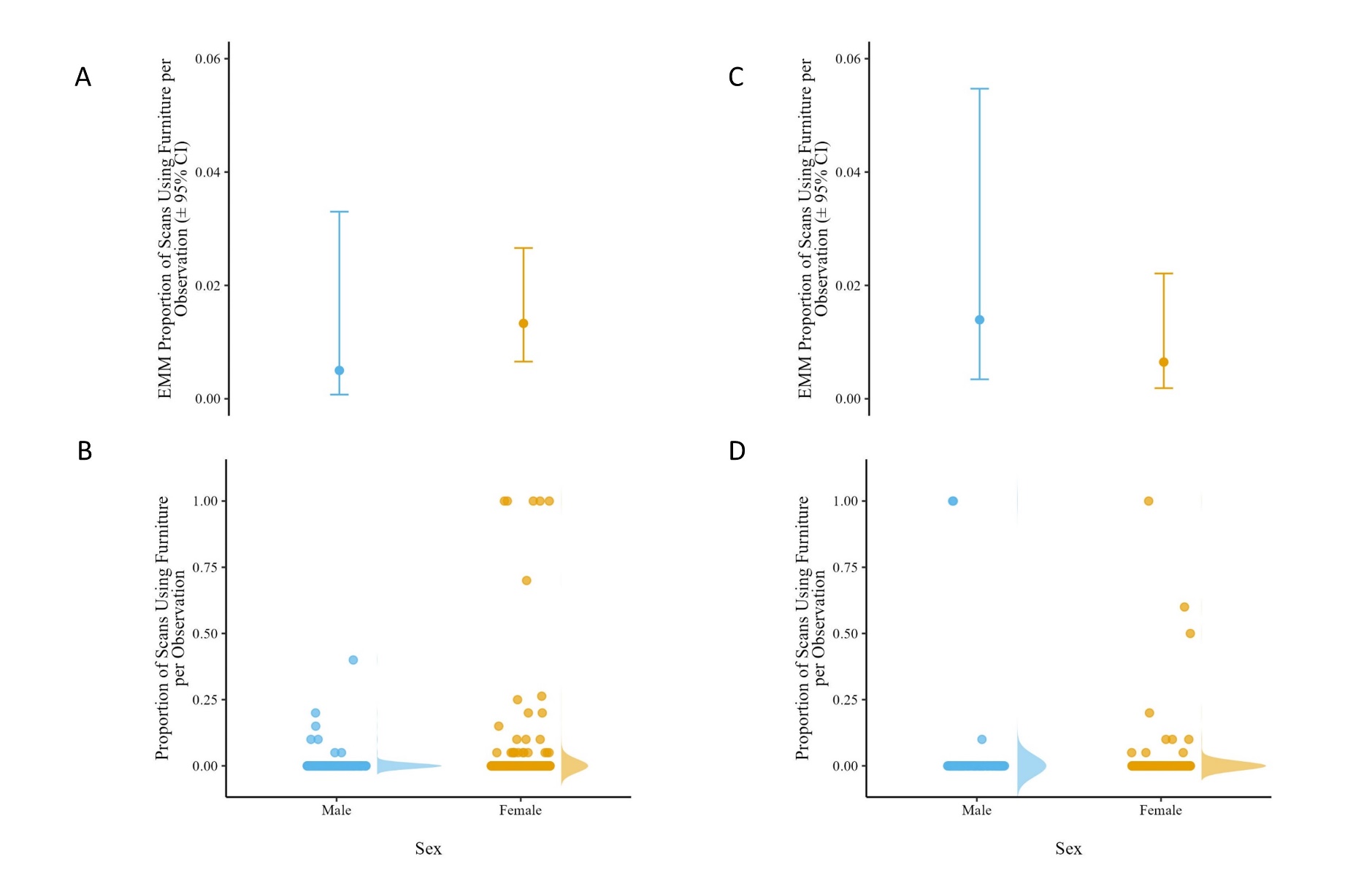
**S14 Figure.** A&C: estimated marginal mean (EMM) of proportion of furniture use behavior per observation by sex bounded by 95% confidence intervals (CI) at night (A) and during the day (C). B&D: proportion of furniture use behavior per observation by sex at night (B) and during the day (D) displayed via dot plot and distribution curve for all observations (n_night_ = 791, n_day_ =531).


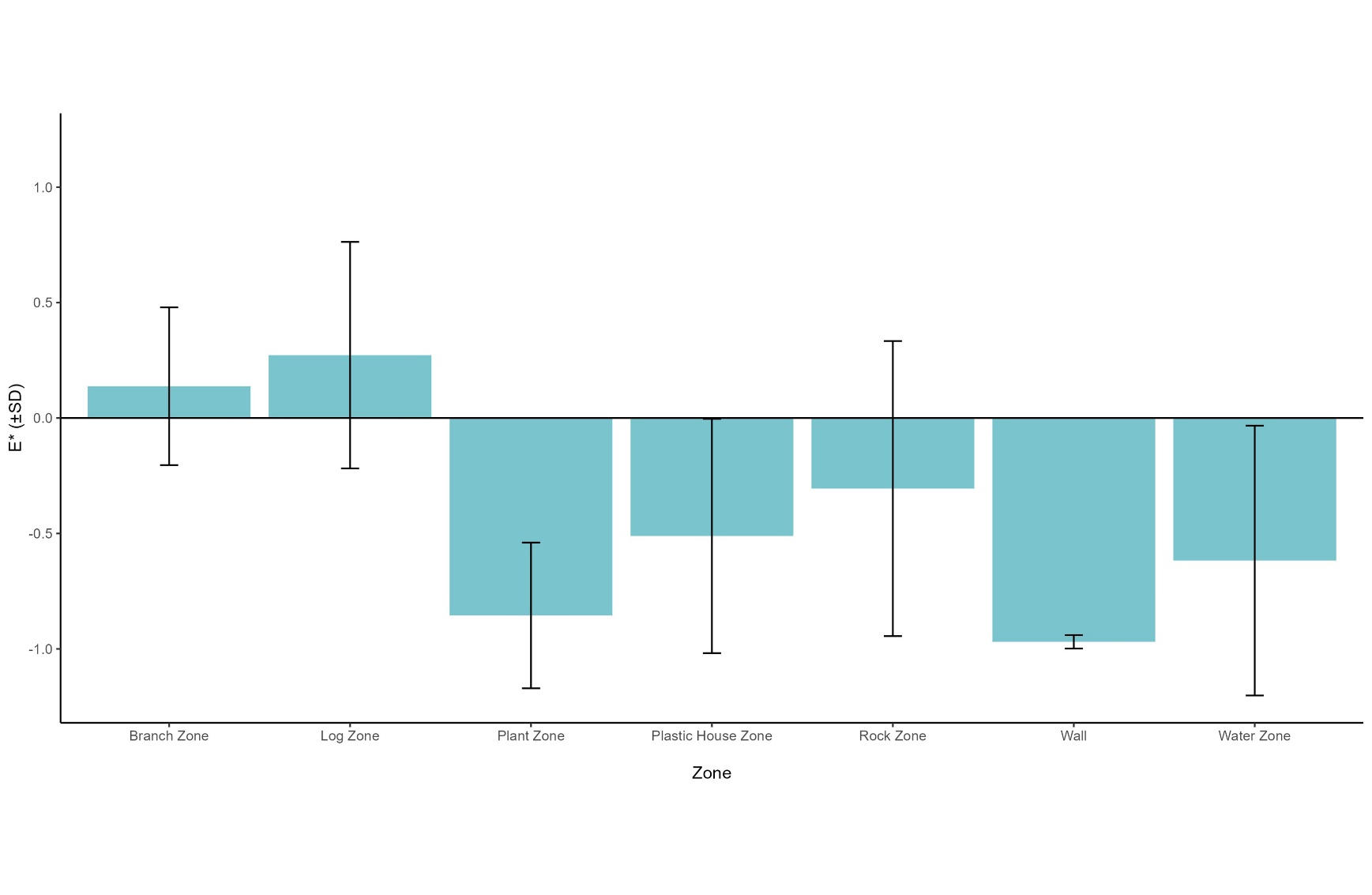
**S15 Figure.** Electivity indices (M±SD) for Complex housing condition by zone.


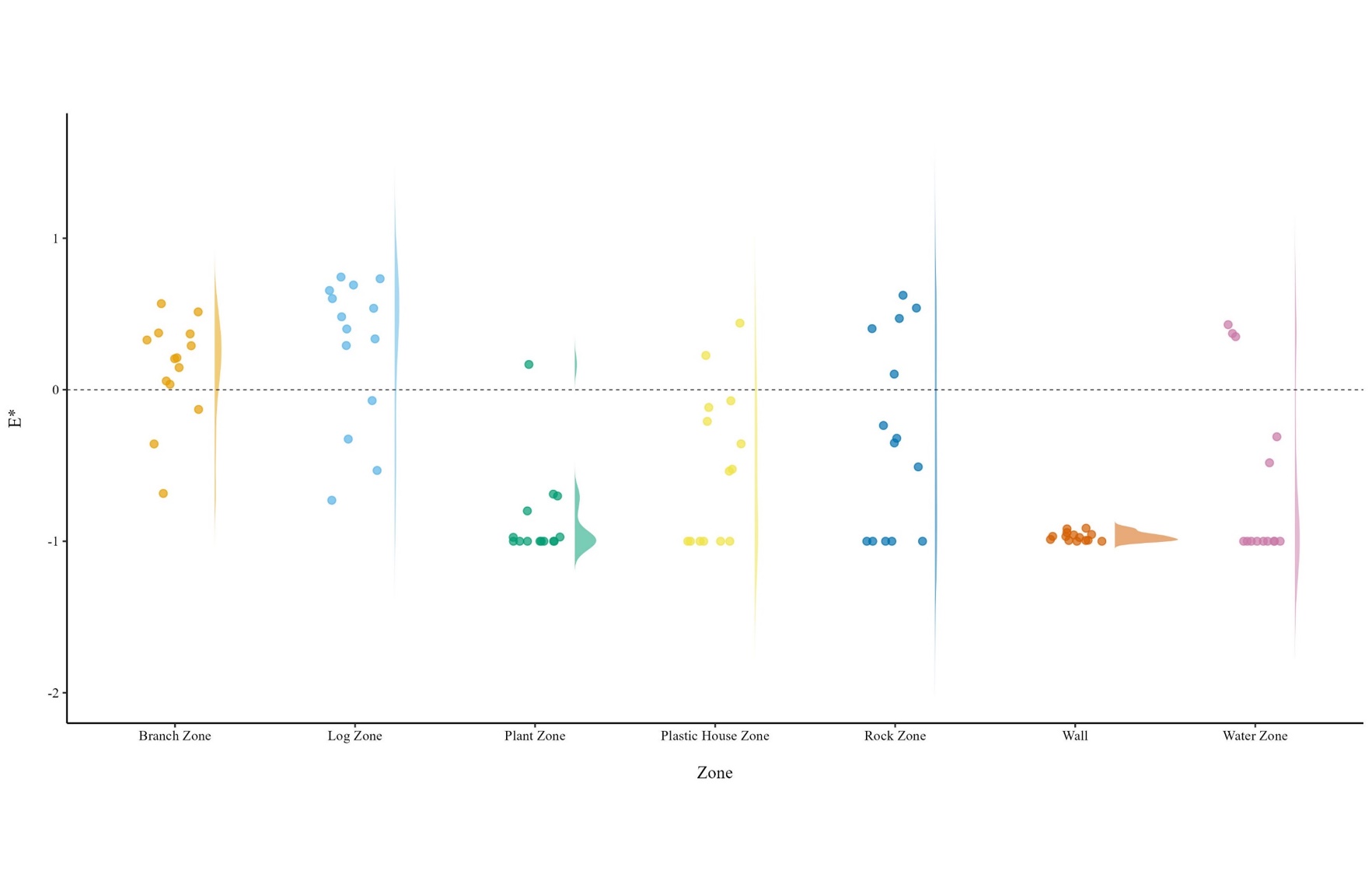
**S16 Figure.** Electivity index values per individual by zone displayed via dot plot for all observations (n = 98).


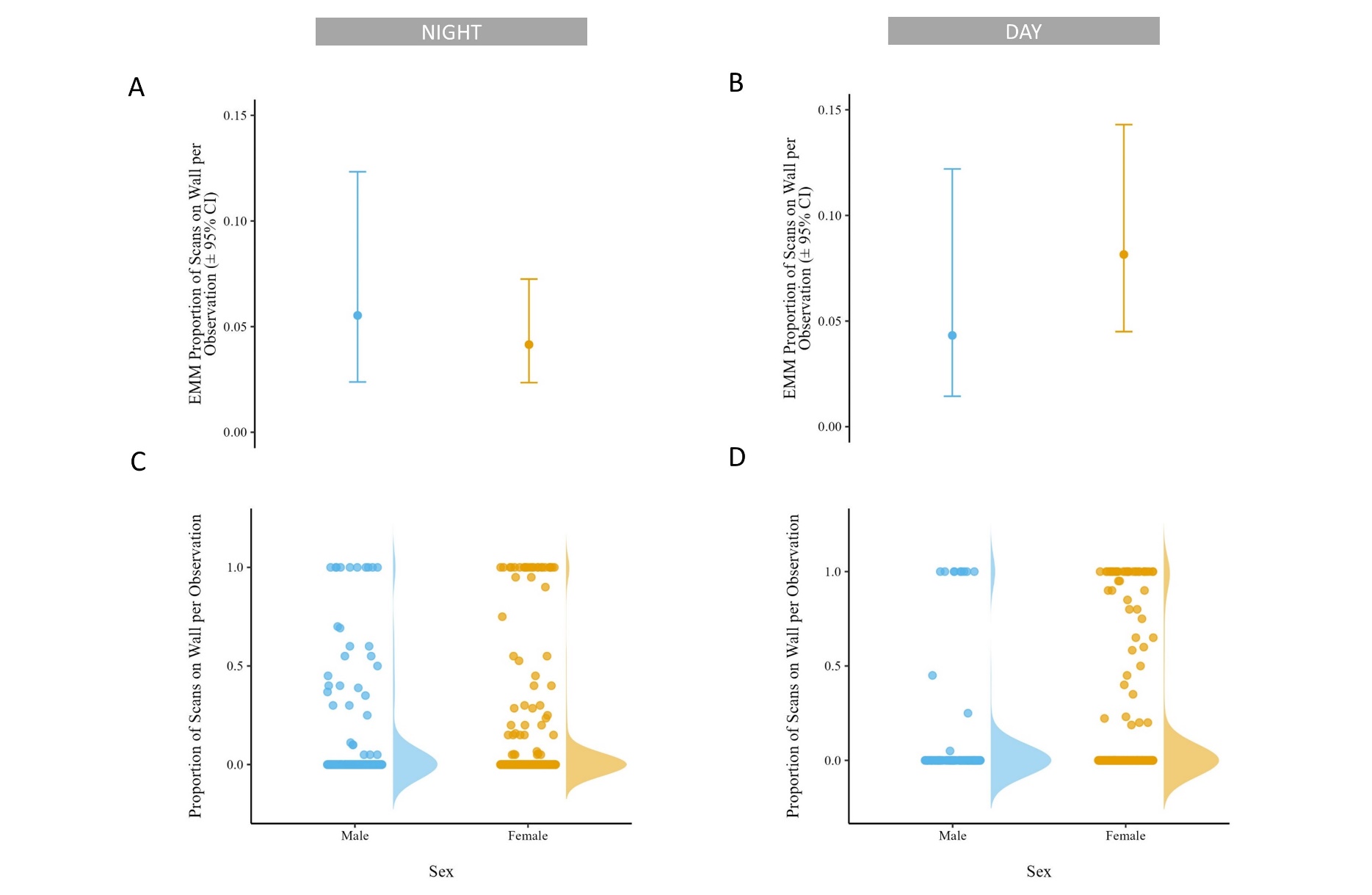
**S17 Figure.** A&C: estimated marginal mean (EMM) of proportion of scans on wall per observation by sex bounded by 95% confidence intervals (CI) at night (A) and during the day (C). B&D: proportion of scans on wall per observation by sex at night (B) and during the day (D) displayed via dot plot and distribution curve for all observations (n_night_ = 739, n_day_ = 486).


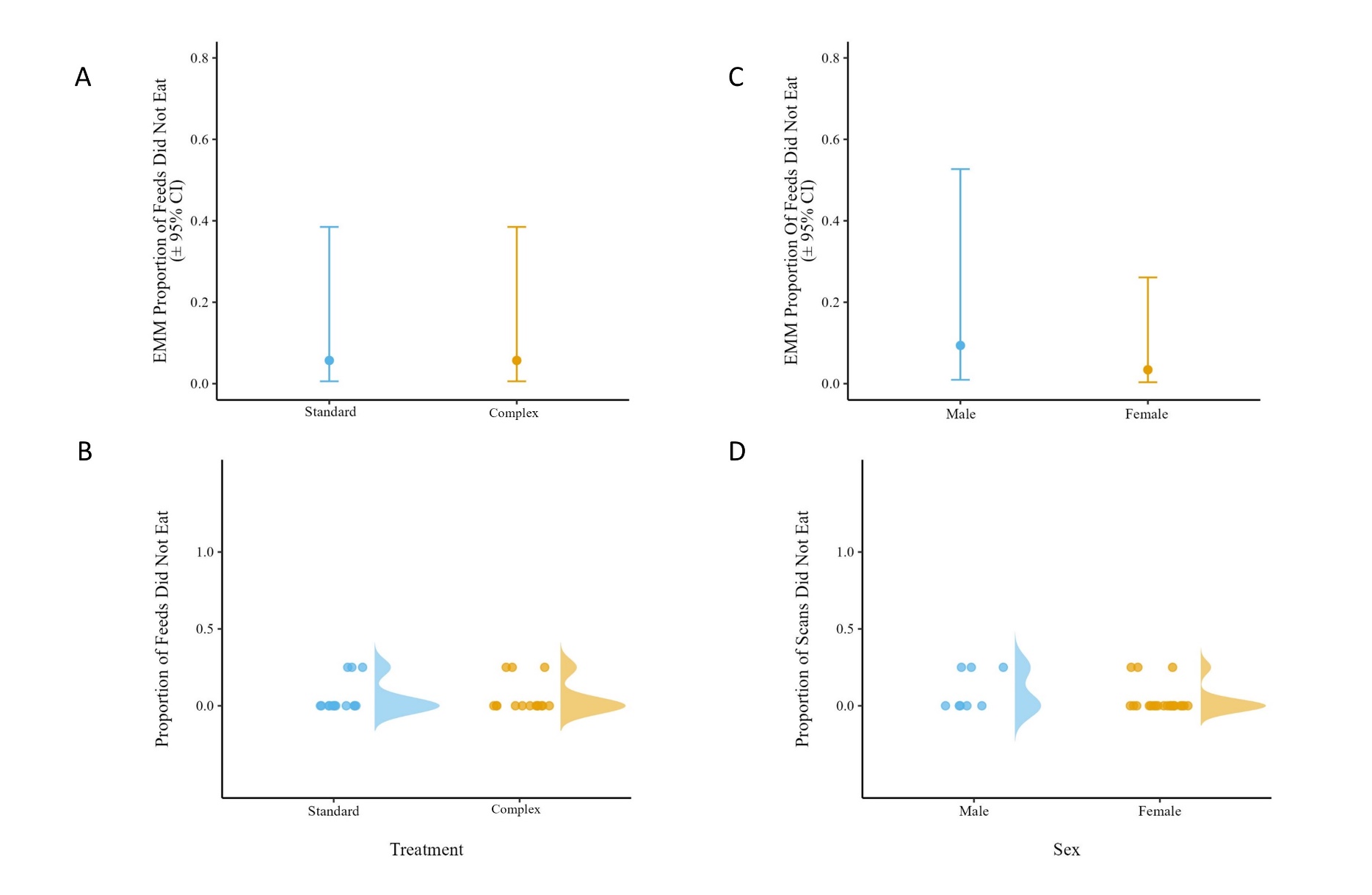
**S18 Figure.** A&C: estimated marginal mean (EMM) of feeds resulting in no food consumption by housing condition (A) and sex (C) with 95% confidence intervals. B&D: proportion of feeds where tarantula did not eat per individual by housing condition (B) and by sex (D) displayed via dot plot and distribution curve for all observations (n = 30).


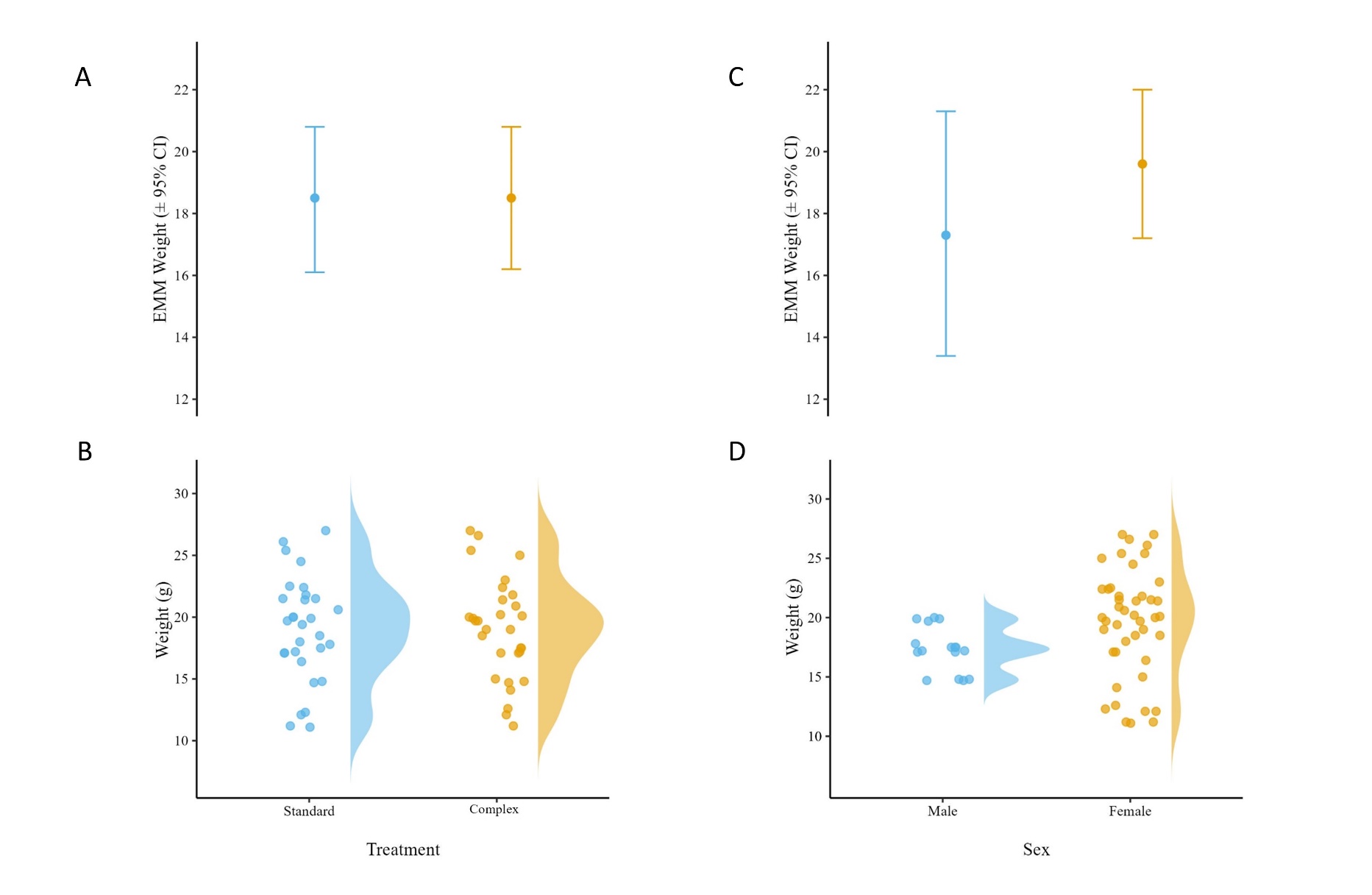
**S19 Figure.** A&C: estimated marginal mean (EMM) of weight per individual by 95% confidence intervals (CI) by housing condition (A) and by sex (C). B&D: weight per individual by housing condition (B) and by sex (D) displayed via dot plot and distribution curve for all observations (n = 60).

**
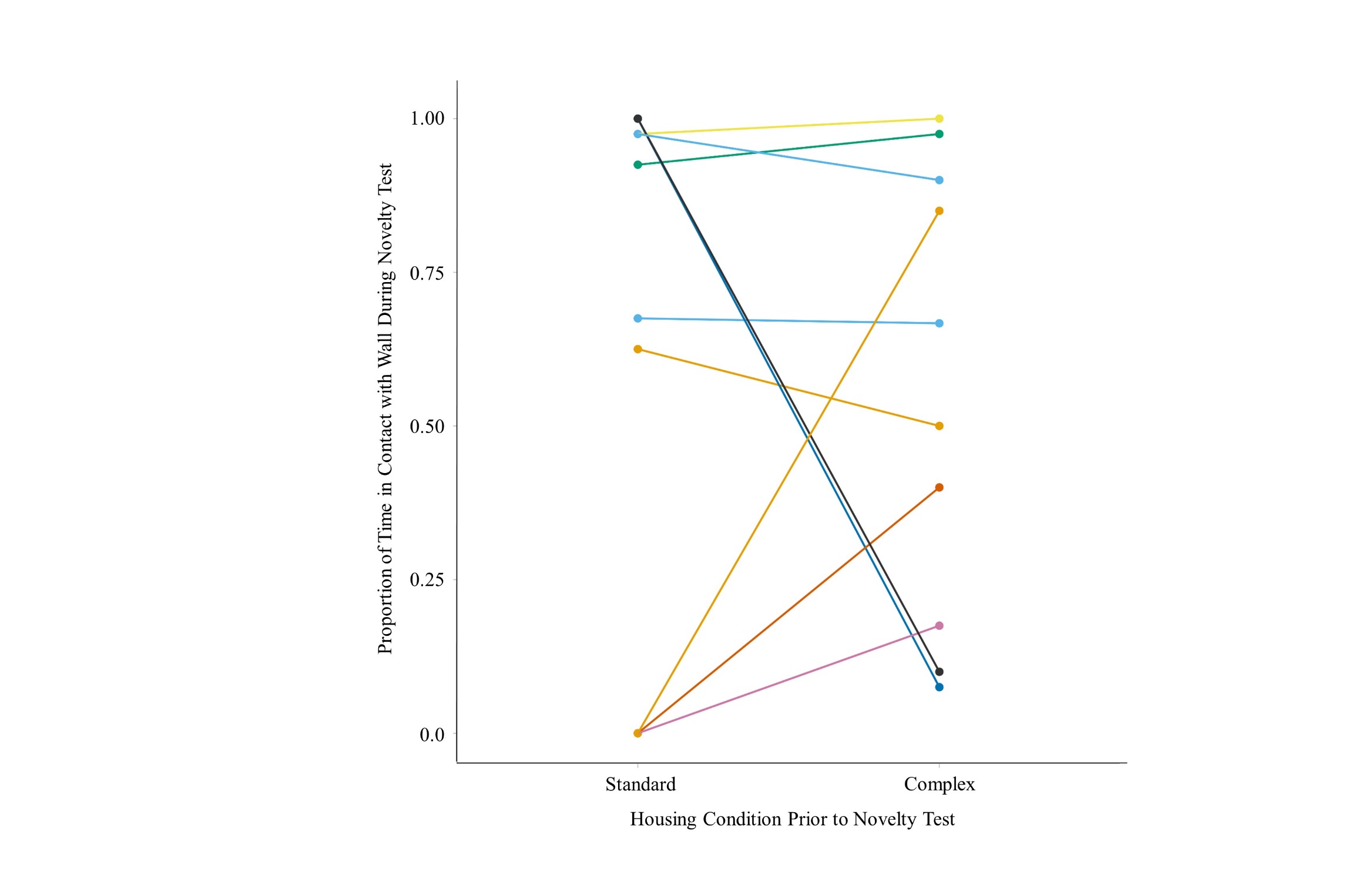
S20 Figure.** Proportion of scans in contact with wall during novelty tests by housing condition.
